# Design and Synthesis of Isosteviol Triazole Conjugates for Cancer Therapy

**DOI:** 10.3390/molecules191118676

**Published:** 2014-11-14

**Authors:** Ravil N. Khaybullin, Mei Zhang, Junjie Fu, Xiao Liang, Tammy Li, Alan R. Katritzky, Paul Okunieff, Xin Qi

**Affiliations:** 1 Department of Medicinal Chemistry, College of Pharmacy, University of Florida, Gainesville, FL 32610, USA; 2 Department of Radiation Oncology, College of Medicine, University of Florida Health Cancer Center, Gainesville, FL 32610, USA; 3 Center for Heterocyclic Compounds, Department of Chemistry, University of Florida, Gainesville, FL 32611, USA

**Keywords:** isosteviol, triazole conjugates, anticancer activity, click chemistry

## Abstract

One of the keys for successfully developing drugs against the broad spectrum of cancer cell types is structural diversity. In the current study, we focused on a family of isosteviol derivatives as potential novel antitumor agents. Isosteviol is a tetracyclic diterpenoid obtained by acid hydrolysis of steviol glycoside extracts isolated from abundant *Stevia rebaudiana* plants. In this work, we have designed and synthesized a panel of isosteviol triazole conjugates using “click” chemistry methodology. Evaluation of these compounds against a series of cancer cell lines derived from primary and metastatic tumors demonstrated that these conjugates exhibit cytotoxic activities with IC_50_ in the low μM range. In addition, their anti-proliferative activities are cancer cell type specific. Taken together, our studies underscore the importance of structural diversity in achieving cancer cell type specific drug development.

## 1. Introduction

Natural products play a significant role in both chemical biology and drug discovery. Their structural complexity enables natural small molecules to aim at a nearly limitless number of biological targets and often do so in a highly selective fashion [1]. Their great structural diversity along with numerous biological characteristics make natural compounds notably attractive both as promising therapeutic agents and a source for searching of new molecular entities with pharmacological activity [1,2,3]. Furthermore, chemical transformation of naturally occurring compounds with known pharmacophore moieties to synthesize hybrid compounds is one of the widely used approaches in medicinal chemistry for obtaining novel therapeutic agents.

**Figure 1:**
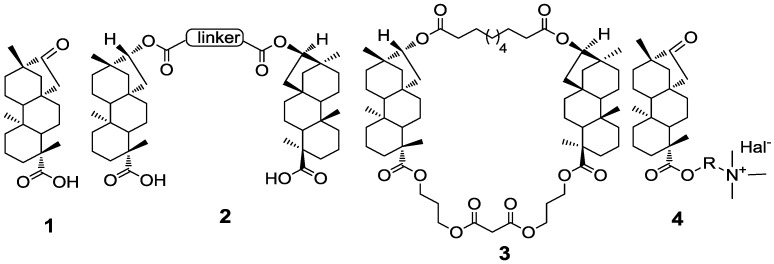
Isosteviol **1** and selected biological active derivatives.

Among the vast pool of natural compounds, diterpenoids, constitute an important class of secondary metabolites that are involved in numerous processes in bio-systems and exhibit considerable pharmacological activities. Nowadays diterpenoid isosteviol **1** (Figure 1) becomes one of the most popular platforms for the design of novel pharmacological agents not only because of its remarkably broad spectrum of biological activities such as antihypertension [4], hypotension [5], antihyperglycaemic [6], cardio [7] and neuroprotective [8] effects, but also due to its extremely easy availability from commercially manufactured glycosides of plant *Stevia Rebaudiana Bertoni*. In recent years, several reviews dealing with chemical transformation and application of isosteviol in organic and medicinal chemistry have been published [9,10,11]. Among the exquisite examples of isosteviol application in a contemporary organic synthesis, synthesis of stereochemically complex templates for small-molecule library [12] and 16-aza-isosteviol derivative as a potential inhibitor of gibberellin biosynthesis in plants [13] demonstrated elegant approaches of chemical transformation.

Notably, some of the synthetic isosteviol derivatives exhibited several new types of biological activities that were not typical for isosteviol. For example, dimer **2** as well as macrocyclic **3** derivatives exhibited tuberculostatic activity [14,15], and ammonium derivatives **4** demonstrated an inhibitive selectivity against acetylcholinesterase [16] (Figure 1).

**Figure 2:**
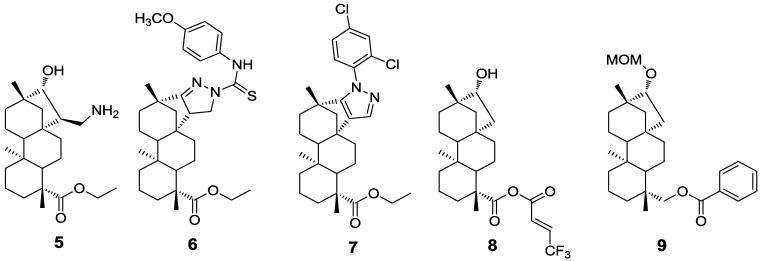
Isosteviol derivatives exerted anti-cancer activity.

Utilizing isosteviol derivatives has a more significant impact on cancer study and treatment. Although some effective chemotherapeutic agents have been developed, they still possess high toxicity and are of relativity high cost. To overcome these challenges, isosteviol represents an attractive scaffold for cancer drug discovery. Indeed, recently published novel cytotoxic isosteviol derivatives starting from commercially available Stevia glycosides have shown promising result. Among them, newly synthesized 1,3-amino alcohol **5** exhibited similar anticancer activities as Cisplatin with IC_50_ value of 4.01 µM [17]. In addition, novel isosteviol-fused pyrazoline and pyrazole derivatives **6**, **7** possess antiproliferative activities on several human malignant cell lines [18] and simple isosteviol derivatives such as esters [19] **8** or MOM-ether [20] **9** are cytotoxic active compounds (Figure 2).

“Click” chemistry is a modern, powerful, reliable, and highly selective technique for the efficient and rapid synthesis of large varieties of complex compounds and bioconjugates. In addition, the triazole scaffold is an attractive moiety not only from a synthetic point of view, but also in the context of biological and drug discovery applications due to its easy formation, planarity with strong dipole moment, and chemical stability alongside with resistance to enzymatic degradation. It was also recently proved that triazole moiety can be recognized as an amide bioisostere [21] and the copper(I)-catalyzed azide-alkyne cycloaddition (CuAAC) has been successfully used for the assembly of unprotected peptide fragments into a bioactive triazole-containing protein. However, only few examples applying “click” chemistry approach to anticancer drug design have been revealed in the literature. Among them, the synthesis of water soluble anthraquinone derivatives [22], bioisosteric analogs of anticancer heterocycles triflorcas [23] and imatinib [24], the triazole analogs of natural products such as geiparvarin [25], diterpenoid dehydroabietic acid [26], and triterpenoid ursolic acid [27] have been reported. In the current report we described the design and synthesis of novel isosteviol conjugates employing “click” chemistry for the purpose of new anticancer drug discovery.

## 2. Results and Discussion

### 2.1. Design and Synthesis of Alkyne Derivatives of Isosteviol.

**Scheme 1:**
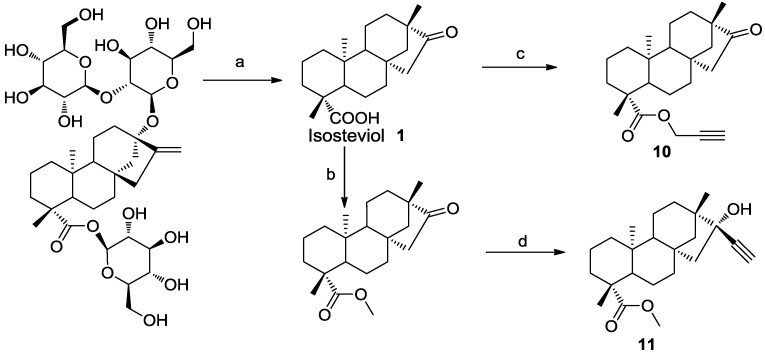
Synthetic alkyne derivatives of isosteviol.

Our starting material, Isosteviol **1**, was readily obtained by acidic hydrolysis of steviol glycosides [28], the commercially available natural sweeteners. On the next step isosteviol core was modified with alkyne functions (derivatives **10** and **11**) to be suitable for “click” reaction. The alkyne **10** was readily obtained by alkylation of isosteviol carboxylic group with propargyl bromide and K_2_CO_3_ in dry acetonitrile while alkyne **11** was prepared from corresponding methyl ester of isosteviol by the Grignard reaction [29]. Nucleophilic addition to the ketone group of the isosteviol always proceeds in high stereoselctive manner. The stereoselectivity can be rationalized by the unique three-dimensional architecture of isosteviol leading to the (R) configuration at the new stereogenic center after attack of the Grignard reagent (Scheme 1).

*Reagents and conditions*: (a) HBr, rt, 24 h, 81%; (b) CH_3_I, K_2_CO_3_, DMF, rt, 85%; (c) 3-bromoprop-1-yne, K_2_CO_3_, CH_3_CN, reflux, 3 h, 97%; (d) ethynylmagnesium bromide, THF, 0 °C, 70%.

### 2.2. Design and Synthesis of the Isosteviol Conjugates

Although procedures for Cu(I)-catalyzed ligation of organic azides and terminal alkynes were well developed, they were not suitable for our desired conjugates. After several attempts, we found the condition for “click” reaction described by Hu [30] as the best for the preparation of isosteviol conjugates with very good yield. Indeed, using CuI(I) and DIPEA/HOAc as the catalytic system, we obtained exclusively 1,4-disubstituted triazoles conjugates without side products.

**Scheme 2:**
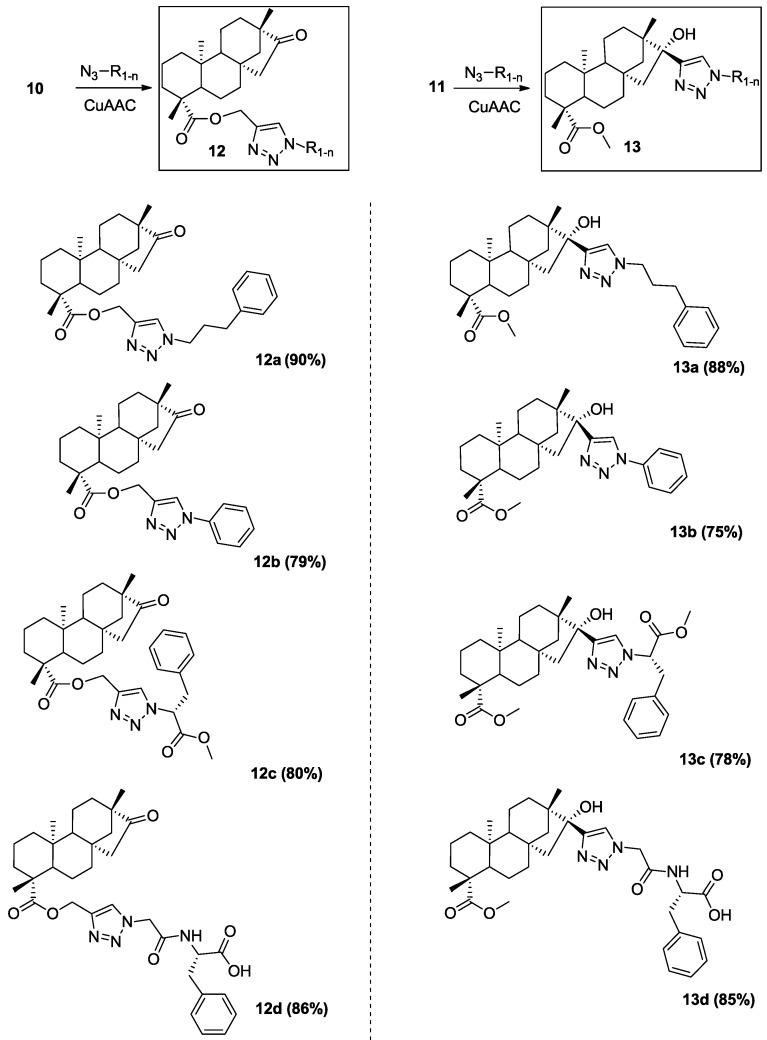
Synthetic isosteviol triazole conjugates.

Both isosteviol alkynes **10** and **11** allow us to design novel isosteviol derivatives with a triazole ring on the different positions of this platform. At the same time, a wide diversity of targeted conjugates was achieved by employing different types of aromatic, aliphatic, amino acid, and peptide azides (Scheme 2). These azides were synthesized following by the procedures reported previously [31,32]. Due to their explosive nature, appropriate safety measures are always taken according to the proper instructions [33,34,35].

All synthesized compounds were characterized by ^1^H-NMR, ^13^C-NMR and HRMS, respectively (all spectra are included in supporting information). According to the NMR analysis, our synthesized triazole conjugates represent exclusively as 1,4-disubstituted triazole regioisomers. Interesting to note, the formation of 1,5-regioisomer was observed only in course of the reaction between sterically hindered alkyl **11** and relatively bulky phenyl azide. The panel of synthetic isosteviol derivatives was divided into two categories. One of them included derivatives obtained from transformation of isosteviol carboxylic group, while the second group of target compounds were ketone-modified derivatives (Scheme 2).

*Reagents and conditions*: Isosteviol alkyne (1 mmol)/azide (1.05 mmol)/CuI (0.05 mmol)/DIPEA (0.05 mmol)/HOAc (0.05 mmol). The yields are given for pure isolated products after column chromatography.

### 2.3. Evaluation of Cytotoxic Activity

The cytotoxic activities of isosteviol derivatives against attached cell lines such as breast (MDA 231), lung (A549), pancreas (ASPC-1), prostate (PC-3), colon (HCT 116) and cervical (HeLa) cancer cell lines were determined by means of 3-(4,5-dimethyl-2-thiazolyl)-2,5-diphenyl-2H-tetrazolium bromide (MTT) assay. Furthermore, the ability of designed compounds to affect proliferations of suspension cell lines such as leukemia cells (MOLT-4 and HL-60) was tested by the CellTiter-Glo^®^ Luminescent Cell Viability Assay to determine the number of viable cells in culture based on quantitation of the ATP present. The IC_50_ values were obtained from fitting data with GraphPad software to determine the growth inhibition in the presence of test compounds.

**Figure 3:**
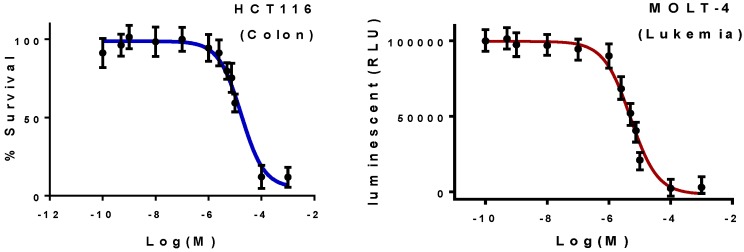
Representative IC_50_ fitting curves: effect of active isosteviol conjugate **12a** on the colon (HCT116) using MTT assay and leukemia (MOLT-4) using CellTiter-Glo^®^ Luminescent Cell Viability Assay.

We started our biological investigation from the study of the ability of these new isosteviol conjugates to inhibit the growth of six different human attached cancer cell lines. Almost all of the tested compounds demonstrated cell growth inhibitory activities (Table 1, Figure 3). Our prior attention was addressed towards the influence of the nature of triazole-containing moieties of synthesized conjugates on their activity. As shown in Table 1, isosteviol conjugate **12a** obtained from alkyne **10** and (3-azidopropyl)benzene exerted significantly more potent anticancer activity than all synthesized derivatives. Interesting to note that compound **12a** demonstrated good activity against all tested cancer cell lines. The linker between triazole heterocycle and benzene ring for the isosteviol carboxylic group modified conjugates played one of the key roles in cytotoxic activity. Indeed, conjugate **12b** without that linker lost antitumor activity in case of lung, pancreas, breast, and prostate cancer cells but still possess good ability to inhibit the growth of colon and cervical cells and so became a more specific agent. The attempt to introduce a polar methoxycarbonyl group into the aliphatic linker between triazole and benzene rings (by reaction of alkyne **10** with L-azidophenylalanine) for conjugate **12c** led to the loss of anticancer activity. Further modification of triazole-contained moiety with peptide fragment (by reaction of alkyne **10** with azidoglycyl-L-phenylalanine) provided us polar conjugates **12d** containing triazole motif with alkylbenzene and linker with free carboxylic group and amide fragment. *In vitro* anticancer study of the compound **12d** revealed no activity against all six human attached tumor cell lines (Table 1). Based on these facts, we can conclude the increasing polarity of triazole-containing moiety significantly decreases anticancer activity of our isosteviol conjugates. At the same time hydrophobicity of triazole moiety plays a key role for their inhibitory activities of isosteviol conjugates to cancer cell growth.

**Table 1:** Cytotoxic activities of isosteviol derivatives against six human attached cancer cell lines ^a^.

Compound	IC_50_ (µM)					
	A549 (Lung)	ASPC-1 (Pancreas)	MDA231 (Breast)	PC-3 (Prostate)	HCT116 (Colon)	HeLa (Cervical)
**12a**	9.95 ± 0.24	4.79 ± 0.18	13.76 ± 0.63	18.23 ± 0.95	6.60 ± 0.38	20.18 ± 1.03
**12b**	98.42 ± 5.63	>100	>100	>100	13.66 ± 0.41	5.83 ± 0.33
**12c**	63.71 ± 3.84	>100	>100	>100	>100	51.14 ± 3.65
**12d**	>100	>100	>100	>100	>100	>100
**13a**	43.52 ± 1.52	>100	69.2 ± 5.23	>100	45.95 ± 2.33	29.62 ± 1.52
**13b**	31.7 ± 1.84	>100	38.12 ± 1.82	73.65 ± 5.61	37.71 ± 1.03	30.72 ± 0.62
**13c**	56.4 ± 1.98	54.68 ± 3.28	50.13 ± 3.08	43.84 ± 1.33	48.9 ± 1.93	40.57 ± 2.81
**13d**	>100	>100	>100	>100	>100	>100

^a^ Cell viability was analyzed by the MTT assay. All measurements were performed in triplicate. Data was represented as mean ± standard deviation (SD).

Our next efforts were focused on examining how the activity will change for isosteviol conjugates baring the same triazole moieties but at different positions of tetracyclic skeleton. For these purposes, the fragments with triazole residue were connected to C-15 position of isosteviol skeleton. Therefore the conjugates **13a**–**d** all bear hydroxyl instead of a ketone functional group at C-15.

The cytotoxic activities for the derivatives **13a**–**d** on attached cancer cell lines were depicted in Table 1. Comparing with the most active compound **12a**, corresponding compound **13a** lost ability to inhibit cell growth after modification of the conjugates structure as described above. Although the inhibitory ability of conjugate **13b** was less potent towards the colon cancer cell line compared with analog **12b**, the compound **13b** demonstrated improved activity towards lung and breast cancer cell lines. The same activity improvements were observed for compound **13c** in comparison to **12c** towards all six attached cancer cell lines. Finally, both conjugates **12d** and **13d** are not active compounds and thus this fact unambiguously indicates that free carboxyl groups within the linker dramatically demolish anticancer activity of these conjugates. All these results are consistent with the facts that ketone group of designed conjugates as well as hydrophobicity of triazole moiety could play key roles in anticancer activity.

Inspired by anticancer study results on the attached tumor cell lines, we further tested the designed compounds towards suspension leukemia human cancer cell lines. Almost all compounds demonstrated improved anti-proliferative activities to inhibit the growth of leukemia tumor cells, MOLT-4 and HL-60 (Table 2, Figure 3). 

**Table 2:** Cytotoxic activities of isosteviol derivatives against human leukemia suspension cancer cell lines ^a^.

Compound	IC_50_ (µM)		Compound	IC_50_ (µM)	
	MOLT-4 (Leukemia)	HL-60 (Leukemia)		MOLT-4 (Leukemia)	HL-60 (Leukemia)
12a	5.02 ± 0.15	28.8 ± 0.63	13a	12.8 ± 0.54	42.38 ± 2.89
12b	7.27 ± 0.32	35.68 ± 1.08	13b	21.04 ± 1.03	63.62 ± 3.02
12c	35.49 ± 0.77	46.77 ± 1.47	13c	31.26 ± 1.28	32.89 ± 1.87
12d	>100	>100	13d	>100	>100

^a^ Cell viability was analyzed by the CellTiter-Glo^®^ Luminescent Cell Viability Assay. All measurements were performed in triplicate. Data was represented as mean ± standard deviation (SD).

The structure-activity-relationship between designed conjugates and cytotoxic activity against leukemia cancer cell lines (Table 2) consistently reveals the similar behavior as in the case of attached tumor cells (Table 1). Thus, the compound **12a** is the most active against both leukemia and the panel of attached cell lines of A549, ASPC-1, MDA231, PC-3, HCT116 and HeLa. The tendency of decreasing anticancer activity along with declining hydrophobicity of triazole moiety remains the same for leukemia cell lines. Compounds **12d** and **13d** bearing free carboxyl group are not active against leukemia cancer cell lines either. In general, the luminescent cell viability data revealed relatively high activity for designed conjugates **12a**, **12b**, and **13a** against MOLT-4 cancer cell line than HL-60 (Table 2).

## 3. Experimental Section

### 3.1. General Procedures

^1^H-NMR and ^13^C-NMR spectra were recorded on a Varian NMR spectrometers operating at 300 MHz or 500 MHz for ^1^H, and 75 MHz or 125 MHz for ^13^C. All chemical shifts were measured in CDCl_3_ and DMSO-d6 as solvents. Melting points were determined on a capillary point apparatus equipped with a digital thermometer and were uncorrected. All chemicals were purchased from Sigma Aldrich (St. Louis, MO, USA) or Acros (Geel, Belgium) and were used as received, unless stated otherwise. High resolution mass spectroscopic data were acquired on an Agilent 6220 ESI-TOF with electro spray ionization (ESI) mode. Reactions were carried out in oven-dried glassware under nitrogen atmosphere, unless otherwise noted. Analytical TLC was performed on E. Merck silica gel 60 F254 plates and visualized by UV and potassium permanganate staining. Flash column chromatography was performed on E. Merck silica gel 60 (40–63 mm). Yields refer to chromatographically and spectroscopically pure compounds.

### 3.2. Synthesis

#### 3.2.1. Preparation of Alkyne Isosteviol (**10**)

*(4R,6aR,9S,11bS)-prop-2-yn-1-yl-4,9,11b-trimethyl-8-oxotetradecahydro-6a,9-methanocyclohepta[a]naphthalene-4-carboxylate* (**10**). Isosteviol (955 mg, 3 mmol), K_2_CO_3_ (829 mg, 6 mmol), and 3-bromoprop-1-yne (580 mg as 80% solution in toluene, 3.9 mmol) were dissolved in 60 mL acetonitrile. After reflux of the above mixture for 3 h, the resulting mixture was cooled to room temperature. The precipitate was then recovered through filtration and washed repeatedly with EtOAc. Combined mother liquids were concentrated under vacuum. Resulting residue was dissolved in EtOAc, washed with 1 N HCl and one more time with water (20 mL). The organic layer was dried over MgSO_4_ and concentrated under reduced pressure. The crude solid product was recrystallized from MeOH to yield 1036 mg (2.91 mmol, 97%) of white crystals of isosteviol propargyl ester (**10**). White solid (97%). ^1^H-NMR (CDCl_3_, 300 MHz, ppm): δ 4.73–4.57 (m, 2H), 2.64 (dd, *J* = 18.6, 3.8 Hz, 1H), 2.45–2.41 (m, 1H), 2.19 (d, *J* = 13.2 Hz, 1H), 1.94–1.22 (m, 13H), 1.21 (s, 3H), 1.20–1.10 (m, 3H), 1.05 (dd, *J* = 13.4, 4.1 Hz, 1H), 0.97 (s, 3H), 0.96–0.84 (m, 1H), 0.73 (s, 3H). ^13^C-NMR (CDCl_3_, 75 MHz, ppm): δ 222.7, 176.5, 77.2, 74.7, 57.2, 54.8, 54.4, 51.6, 48.9, 48.6, 44.1, 41.6, 39.9, 39.6, 38.2, 38.1, 37.5, 28.9, 21.8, 20.5, 20.0, 19.0, 13.7. HRMS: calcd. for C_23_H_33_O_3_ [M+H]^+^ 357.2424, found 357.2423. MP 115–116 °C.

#### 3.2.2. General Procedures for the Preparation of Conjugates by Copper(I)-catalyzed Azide-alkyne Cycloaddition Reaction

To a solution of an alkyne (1 mmol), an azide (1.05 mmol), DIPEA (0.05 mmol) and HOAc (0.05 mmol) in CH_2_Cl_2_ (5 mL) was added CuI (0.05 mmol) under nitrogen atmosphere at room temperature. Reaction mixture was stirred at room temperature until alkyne was consumed (TLC control). The reactions mixture was diluted with DCM and washed 1 times with 1 N HCl, water, and dried over MgSO_4_. The crude product obtained after evaporation of solvent was purified by flash column chromatography on silica gel. Eluents: Hexane/EtOAc = 2:1 (for compounds **12a**, **12c**, **13a**, **13b**, **13c**); DCM/MeOH = 130:1 (for compound **12b**); EtOAc/MeOH = 3:1 (for compound **13d**); DCM/MeOH = 25:1 (for compound **12d**).

*(4R,6aR,9S,11bS)-(1-(3-Phenylpropyl)-1H-1,2,3-triazol-4-yl)methyl-4,9,11b-trimethyl-8-oxotetradecahydro-6a,9-methanocyclohepta[a]naphthalene-4-carboxylate* (**12a**). White solid (90%). ^1^H-NMR (CDCl_3_, 300 MHz, ppm): δ 7.55 (s, 1H), 7.36–7.12 (m, 3H), 5.39–5.08 (m, 2H), 4.35 (t, *J* = 7.1 Hz, 2H), 2.64 (t, *J* = 7.5 Hz, 2H), 2.55 (dd, *J* = 18.6, 3.7 Hz, 1H), 2.31–2.20 (m, 2H), 2.19–2.13 (m, 1H), 1.92–1.19 (m, 15 H), 1.17 (s, 3H), 1.16–1.08 (m, 2H), 1.03 (dd, *J* = 13.6, 4.1 Hz, 1H), 0.96 (s, 3H), 0.95–0.81 (m, 1H), 0.54 (s, 3H). ^13^C-NMR (CDCl_3_, 75 MHz, ppm): 222.3, 177.2, 142.7, 140.0, 128.6, 128.4, 128.4, 126.4, 123.8, 57.2, 57.1, 57.1, 54.6, 54.2, 49.5, 48.6, 48.4, 43.9, 41.4, 39.7, 39.4, 38.0, 37.8, 37.2, 32.4, 31.6, 28.9, 21.7, 20.3, 19.8, 18.9, 13.3. HRMS (ESI^+^) calcd for C_32_H_44_N_3_O_3_ [M+H]^+^ 518.3377, found: 518.3383. MP 147–148 °C.

*(4R,6aR,9S,11bS)-(1-Phenyl-1H-1,2,3-triazol-4-yl)methyl4,9,11b-trimethyl-8-oxotetradecahydro-6a,9-methanocyclohepta[a]naphthalene-4-carboxylate* (**12b**). White solid (79%). ^1^H-NMR (CDCl_3_, 300 MHz, ppm): δ 8.01 (s, 1H), 7.83–7.65 (m, 2H), 7.54 (t, *J* = 7.5 Hz, 2H), 7.48–7.42 (m, 1H), 5.38–5.18 (m, 2H), 2.56 (dd, *J* = 18.6, 3.7 Hz, 1H), 2.20 (dd, *J* = 13.7, 2.0 Hz, 1H), 1.94–1.21 (m, 16H), 1.19 (s, 3H), 1.18–1.09 (m, 3H), 1.08–0.97 (m, 1H), 0.96 (s, 3H), 0.88 (td, *J* = 13.1, 3.5 Hz, 1H), 0.58 (s, 3H). ^13^C-NMR (CDCl_3_, 75 MHz, ppm): 222.4, 177.2, 143.4, 136.9, 129.8, 128.9, 122.1, 120.5, 120.5, 57.2, 57.1, 54.7, 54.2, 48.7, 48.4, 43.9, 41.4, 39.7, 39.4, 37.9, 37.2, 28.9, 21.7, 20.3, 19.8, 18.9, 13.3. HRMS (ESI^+^) calcd for C_29_H_38_N_3_O_3_ [M+H]^+^ 476.2908, found: 476.2906. MP 130–131 °C.

*(4R,6aR,9S,11bS)-(1-((R)-1-Methoxy-1-oxo-3-phenylpropan-2-yl)-1H-1,2,3-triazol-4-yl)methyl 4,9,11b-trimethyl-8-oxotetradecahydro-6a,9-methanocyclohepta[a]naphthalene-4-carboxylate* (**12c**). White solid (80%). ^1^H-NMR (CDCl_3_, 300 MHz, ppm): 7.64 (s, 1H), 7.10–6.96 (m, 2H), 5.57 (dd, *J* = 8.9, 6.1 Hz, 1H), 5.16 (s, 2H), 3.75 (s, 3H), 3.60–3.29 (m, 2H), 2.54 (dd, *J* = 18.7, 3.7 Hz, 3H), 2.16 (d, *J* = 13.3 Hz, 1H), 1.91–1.53 (m, 9H), 1.49 (dd, *J* = 8.5, 3.5 Hz, 1H), 1.45–1.16 (m, 6H), 1.13 (s, 3H), 1.13–1.06 (m, 1H), 1.02 (dd, *J* = 13.5, 4.2 Hz, 1H), 0.96 (s, 3H), 0.94–0.79 (m, 1H), 0.53 (s, 3H). ^13^C-NMR (CDCl_3_, 75 MHz, ppm): 222.6, 177.2, 168.6, 142.8, 134.7, 128.9, 127.7, 124.1, 64.1, 57.2, 57.2, 54.8, 54.4, 53.3, 48.8, 48.5, 44.0, 41.6, 39.8, 39.5, 38.8, 38.1, 38.0, 37.4, 29.0, 21.8, 20.4, 20.0, 19.0, 13.5. HRMS (ESI^+^) calcd for C_33_H_44_N_3_O_5_ [M+H]^+^ 562.3275, found: 562.3276. MP 67–69 °C.

*(2S)-3-Phenyl-2-(2-(4-((((4R,6aR,9S,11bS)-4,9,11b-trimethyl-8-oxotetradecahydro-6a,9-methanocyclohepta[a]naphthalene-4-carbonyl)oxy)methyl)-1H-1,2,3-triazol-1-yl)acetamido)propanoic acid* (**12d**). White solid (86%). ^1^H-NMR (CDCl_3_, 300 MHz, ppm): 7.77 (s, 1H), 7.36–7.12 (m, 5H), 6.76–6.49 (m, 1H), 5.17 (dd, *J* = 47.1, 12.6 Hz, 2H), 4.98 (dd, *J* = 71.2, 16.0 Hz, 2H), 4.83–4.73 (m, 1H), 3.29–3.18 (m, 1H), 3.12–3.01 (m, 1H), 2.72 (dd, *J* = 19.0, 3.6 Hz, 1H), 2.24–2.12 (m, 1H), 1.91–1.24 (m, 14H), 1.19 (s, 3H), 1.16–0.99 (m, 4H), 0.97 (s, 3H), 0.82 (td, *J* = 13.6, 4.5 Hz, 1H), 0.25 (s, 3H). ^13^C-NMR (CDCl_3_, 75 MHz, ppm): 226.2, 176.9, 172.7, 164.8, 142.9, 135.8, 129.3, 128.6, 127.2, 125.9, 60.4, 57.2, 56.8, 54.4, 54.0, 53.6, 52.5, 49.0, 48.1, 43.8, 41.2, 39.6, 37.9, 37.7, 37.5, 37.1, 29.1, 21.5, 21.0, 20.2, 19.7, 18.9, 14.2, 13.0. HRMS (ESI^+^) calcd for C_34_H_44_N_4_O_6_ [M+H]^+^ 605.3334, found: 605.3343. MP 127–128 °C.

*(4R,6aR,8R,9S,11bS)-Methyl-8-hydroxy-4,9,11b-trimethyl-8-(1-(3-phenylpropyl)-1H-1,2,3-triazol-4-yl)tetradecahydro-6a,9-methanocyclohepta[a]naphthalene-4-carboxylate* (**13a**). White solid (88%). ^1^H-NMR (CDCl_3_, 300 MHz, ppm): 7.37–7.11 (m, 6H), 4.32 (t, *J* = 7.0 Hz, 2H), 3.60 (s, 3H), 3.05 (s, 1H), 2.63 (t, *J* = 7.5 Hz, 2H), 2.39 (dd, *J* = 14.7, 2.5 Hz, 1H), 2.33–1.22 (m, 19H), 1.17 (s, 3H), 1.14–0.99 (m, 5H), 0.93–0.81 (m, 1H), 0.76 (s, 1H), 0.43 (s, 1H). ^13^C-NMR (CDCl_3_, 75 MHz, ppm): 178.1, 156.0, 140.1, 128.6, 128.4, 126.3, 120.2, 81.6, 57.1, 55.8, 54.8, 51.1, 51.1, 50.6, 49.4, 45.9, 43.8, 41.6, 41.2, 39.9, 38.0, 36.3, 32.4, 31.7, 28.9, 22.4, 21.8, 20.8, 18.9, 13.1. HRMS (ESI^+^) calcd for C_32_H_46_N_3_O_3_ [M+H]^+^ 520.3534, found: 520.3557. MP 76–78 °C.

*(4R,6aR,8R,9S,11bS)-Methyl-8-hydroxy-4,9,11b-trimethyl-8-(1-phenyl-1H-1,2,3-triazol-4-yl)tetradecahydro-6a,9-methanocyclohepta[a]naphthalene-4-carboxylate* (**13b**). White solid (75%). ^1^H-NMR (CDCl_3_, 300 MHz, ppm): 7.80 (s, 1H), 7.79–7.71 (m, 2H), 7.59–7.37 (m, 3H), 3.61 (s, 3H), 2.42 (dd, *J* = 14.7, 2.6 Hz, 1H), 2.27–2.12 (m, 2H), 2.02–1.28 (m, 14H), 1.18 (s, 3H), 1.17–1.04 (m, 4H), 1.04–0.84 (m, 2H), 0.77 (s, 3H), 0.51 (s, 3H). ^13^C-NMR (CDCl_3_, 75 MHz, ppm): 178.1, 156.8, 137.1, 129.7, 129.7, 128.6, 120.4, 118.4, 110.0, 81.8, 60.4, 57.1, 55.8, 54.8, 51.1, 50.5, 46.0, 43.8, 41.5, 41.3, 39.9, 38.0, 36.3, 28.9, 22.4, 21.8, 20.8, 18.9, 14.2, 13.2. HRMS (ESI^+^) calcd for C_29_H_40_N_3_O_3_ [M+H]^+^ 478.3070, found: 478.3063. MP 119–122 °C.

*(4R,6aR,8R,9S,11bS)-Methyl 8-hydroxy-8-(1-((S)-1-methoxy-1-oxo-3-phenylpropan-2-yl)-1H-1,2,3-triazol-4-yl)-4,9,11b-trimethyltetradecahydro-6a,9-methanocyclohepta[a]naphthalene-4-carboxylate* (**13c**). White solid (75%). ^1^H-NMR (CDCl_3_, 500 MHz, ppm): 7.30 (s, 1H), 7.27–7.17 (m, 3H), 7.08–6.98 (m, 2H), 5.61–5.48 (m, 1H), 3.78 (s, 3H), 3.62 (s, 3H), 3.58–3.45 (m, 2H), 2.98 (s, 1H), 2.37 (dd, *J* = 15.0, 2.7 Hz, 1H), 2.26–2.13 (m, 1H), 2.02 (d, *J* = 14.7 Hz, 1H), 1.98–1.90 (m, 1H), 1.89–1.22 (m, 14H), 1.19 (s, 3H), 1.17–0.85 (m, 6H), 0.76 (s, 3H), 0.33 (s, 3H). ^13^C-NMR (CDCl_3_, 125 MHz, ppm): 178.1, 168.7, 155.8, 134.8, 129.0, 128.8, 127.5, 120.6, 81.5, 63.9, 57.1, 55.8, 54.8, 53.1, 51.1, 50.3, 46.0, 43.8, 41.5, 41.2, 39.9, 38.6, 38.1, 38.0, 36.3, 28.9, 22.2, 21.8, 20.8, 18.9, 13.1. HRMS (ESI^+^) calcd for C_33_H_45_N_3_O_5_ [M+H]^+^ 564.3432, found: 564.3445. MP 144–145 °C.

*(2S)-2-(2-(4-((4R,6aR,8R,9S,11bS)-8-Hydroxy-4-(methoxycarbonyl)-4,9,11b-trimethyltetradecahydro-6a,9-methanocyclohepta[a]naphthalen-8-yl)-1H-1,2,3-triazol-1-yl)acetamido)-3-phenylpropanoic acid* (**13d**). White solid (85%). ^1^H-NMR (DMSO-d6, 500 MHz, ppm): δ 8.54 (s, 1H), 7.67 (s, 1H), 7.45–6.83 (m, 5H), 5.15–4.98 (m, 1H), 4.39 (br s, 1H), 3.55 (s, 3H), 3.01 (ddd, *J* = 91.4, 13.9, 6.7 Hz, 2H), 2.23–2.08 (m, 1H), 2.06 (d, *J* = 13.0 Hz, 1H), 1.95–1.18 (m, 16H), 1.13 (s, 3H), 1.10–0.79 (m, 8H), 0.69 (s, 3H), 0.33 (s, 3H). ^13^C-NMR (DMSO-d6, 125 MHz, ppm): 177.6, 173.4, 167.4, 165.7, 165.6, 156.8, 138.3, 132.2, 132.0, 129.7, 129.1, 128.6, 126.7, 123.7, 80.7, 67.9, 56.7, 55.8, 54.8, 54.5, 51.5, 49.8, 45.7, 43.6, 41.5, 41.3, 38.6, 38.0, 37.9, 37.5, 36.6, 30.3, 28.9, 28.8, 23.7, 22.9, 22.0, 20.8, 19.0, 14.4, 13.4, 11.3. HRMS (ESI^−^) calcd for C_34_H_45_N_4_O_6_ [M-H]^−^ 605.3345, found: 605.3354. MP 223–224 °C.

### 3.3. Biological Materials

Dimethyl sulfoxide (DMSO), 3-(4,5-dimethyl-2-thiazolyl)-2,5-diphenyl-2H-tetrazolium bromide (MTT), and other chemical reagents were purchased from Sigma-Aldrich (St. Louis, MO, USA). CellTiter-Glo^®^ Luminescent Cell Viability Assay was purchased from Promega (Madison, WI, USA). Dulbecco’s Modified Eagle Medium (DMEM), RPMI 1640 medium, and fetal bovine serum (FBS) were purchased from Gibco (Grand Island, NE, USA). All cell lines were purchased from ATCC (Manassas, VA, USA).

### 3.4. Cell Culture and MTT Assay

The anti-proliferative activities of the isosteviol triazole compounds were assessed by the tetrazolium-based MTT assay. Human lung cancer A549 cell line, human pancreatic cancer ASPC-1 cell line, human prostate cancer PC-3 cell line, human colorectal carcinoma HCT-116 cell line, and human breast carcinoma MCF-7 cell line were cultured in DMEM medium supplied with 10% FBS. Cells were seeded in 96 well plates at the density of 5000, 8000, 8000, 12,000 and 6000 cells per well, respectively. Cancer cells were treated with respective compounds for 72 h and then incubated with 100 µL of 0.5 mg/mL 3-(4,5-Dimethyl-2-thiazolyl)-2,5-diphenyl-2H-tetrazolium bromide (MTT) solution for 4 h. The supernatant was discarded and DMSO was added to each well. Absorbance at 570 nm was measured using a SpectraMax M2 reader (Molecular Devices, Sunnyvale, CA, USA). The number of viable cells in the control group was assigned a relative value of 100%.

### 3.5. Cell Titer Glo Assay

The leukemia cell lines MOLT-4 was cultured in RPMI-1640 medium and HL-60 was cultured in DMEM medium, 10% FBS was supplied to both culture systems. For the growth assays, MOLT-4 cell and HL-60 were plated in 96-well plates at 9000 cells/well and 10,000 cells/well. Cells were treated with isostevol triazole compounds at various concentrations. On Day 3, cells were lysed with CellTiter-Glo^®^ Luminescent Cell Viability Assay reagent (Promega) following manufacture’s instruction and luminescence was read using the PerkinElmer Victor^3^ V plate reader (PerkinElmer, Waltham, MA, USA). Percent cell growth was calculated relative to control cells. 

## 4. Conclusions

In summary, we have developed an efficient method for the synthesis of novel isosteviol triazole conjugates using “click” chemistry methodology as cancer therapeutics. The ketone group at C-15 of designed conjugates, polarity of triazole-linker moiety as well as hydrophobicity of triazole moiety all play key roles in anticancer activity of these compounds. The resulting conjugates exert potent anti-proliferative activities with promising selectivity towards a panel of human cancer cell lines.

## Supplementary Materials

Supplementary materials can be accessed at: http://www.mdpi.com/1420-3049/19/11/18676/s1.

## Supplementary Files

Supplementary File 1
