# Supplementary material for: Design and Synthesis of Isosteviol Triazole Conjugates for Cancer Therapy
Source: Molecules. 2014 Nov 14;19(11):18676–89. doi: 10.3390/molecules191118676 (PMC5753759; doi:10.3390/molecules191118676)

Supporting Information

General Procedures

^{1}H-NMR and ^{13}C-NMR spectra were recorded on a Varian NMR spectrometers operating at 300 MHz or 500 MHz for ^{1}H, and 75 MHz or 125 MHz for ^{13}C. All chemical shifts were measured in $CDCl_3$ and DMSO-d6 as solvents. High resolution mass spectroscopic data were acquired on an Agilent 6220 ESI-TOF with electro spray ionization (ESI) mode.

Figure S1. ^{1}H-NMR of compound **12a**.

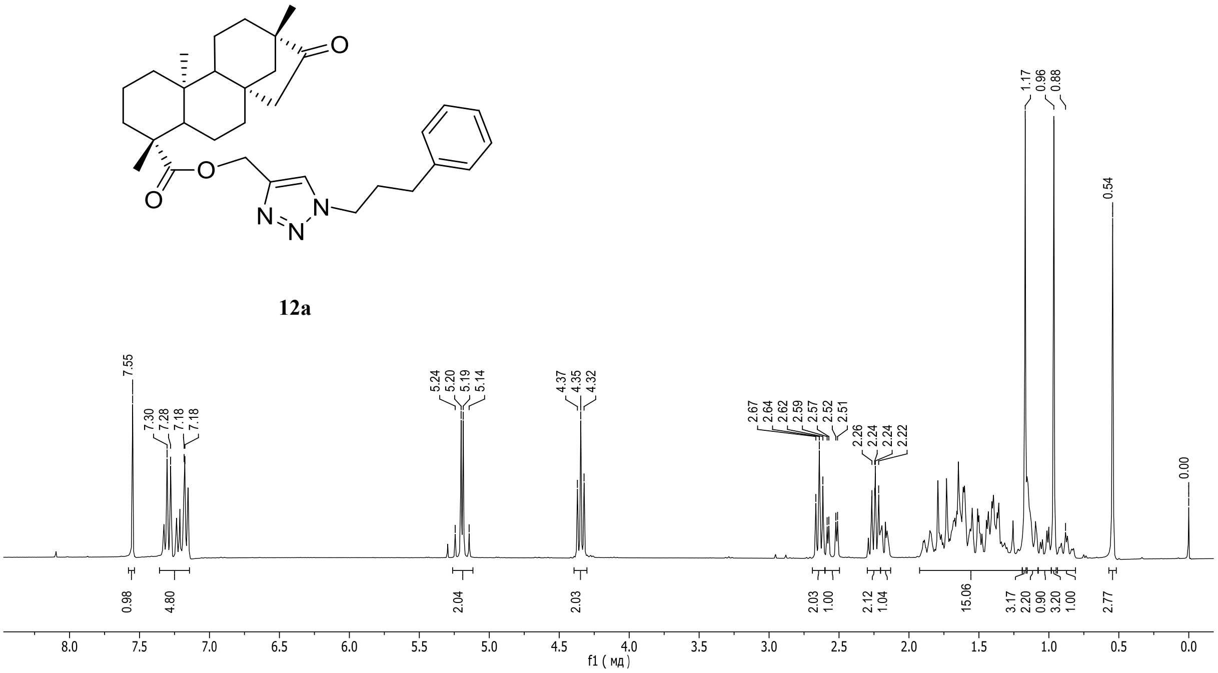

Figure S2. ^{13}C-NMR of compound **12a**.

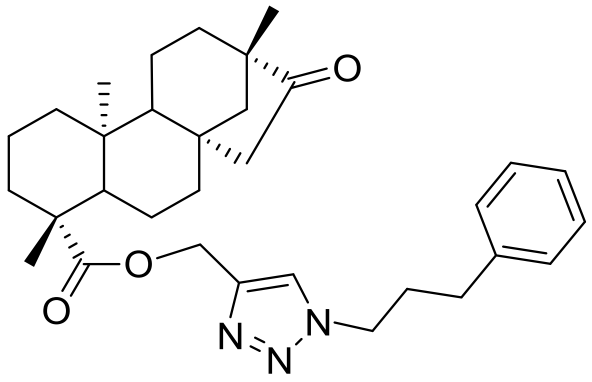

12a

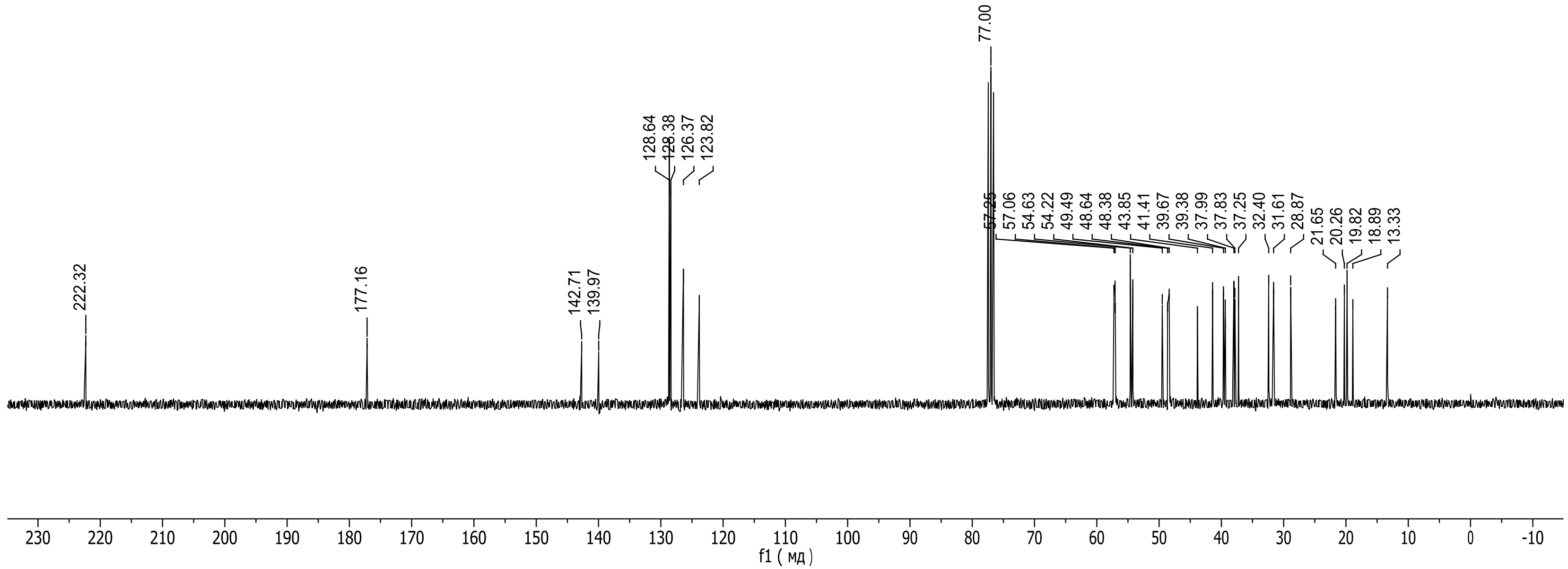

Figure S3. HRMS of compound **12a**.

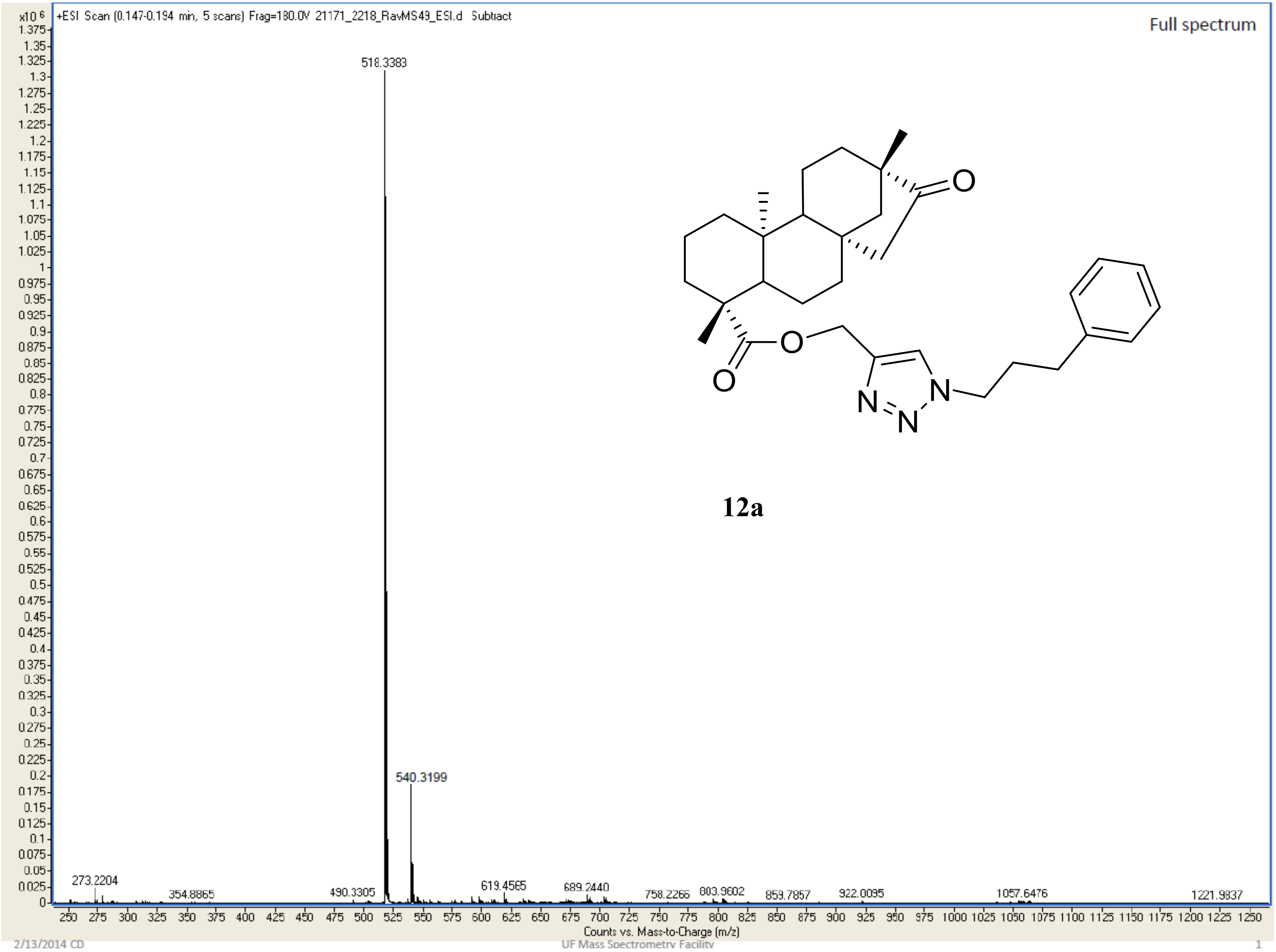

Figure S4. ^{1}H-NMR of compound **12b**.

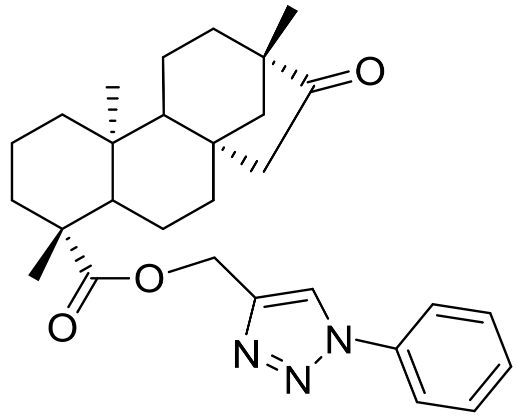

12b

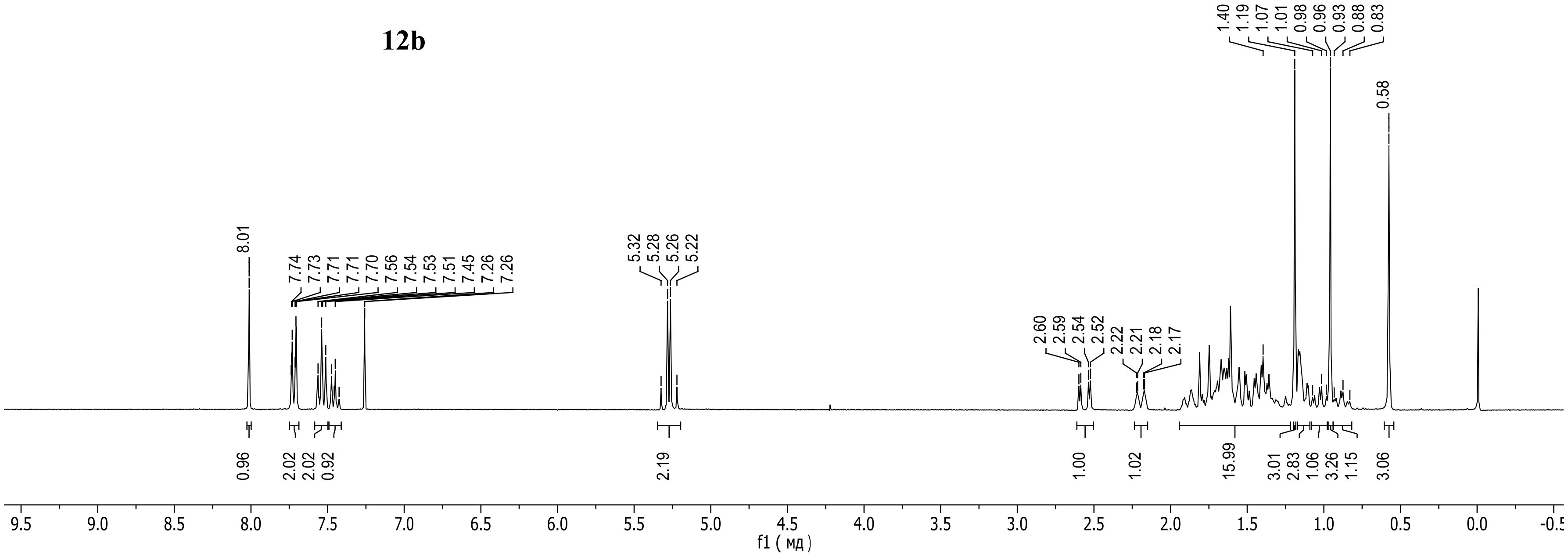

Figure S5. ^{13}C-NMR of compound **12b**.

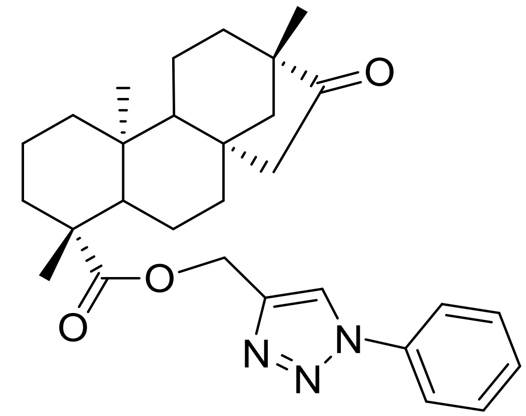

12b

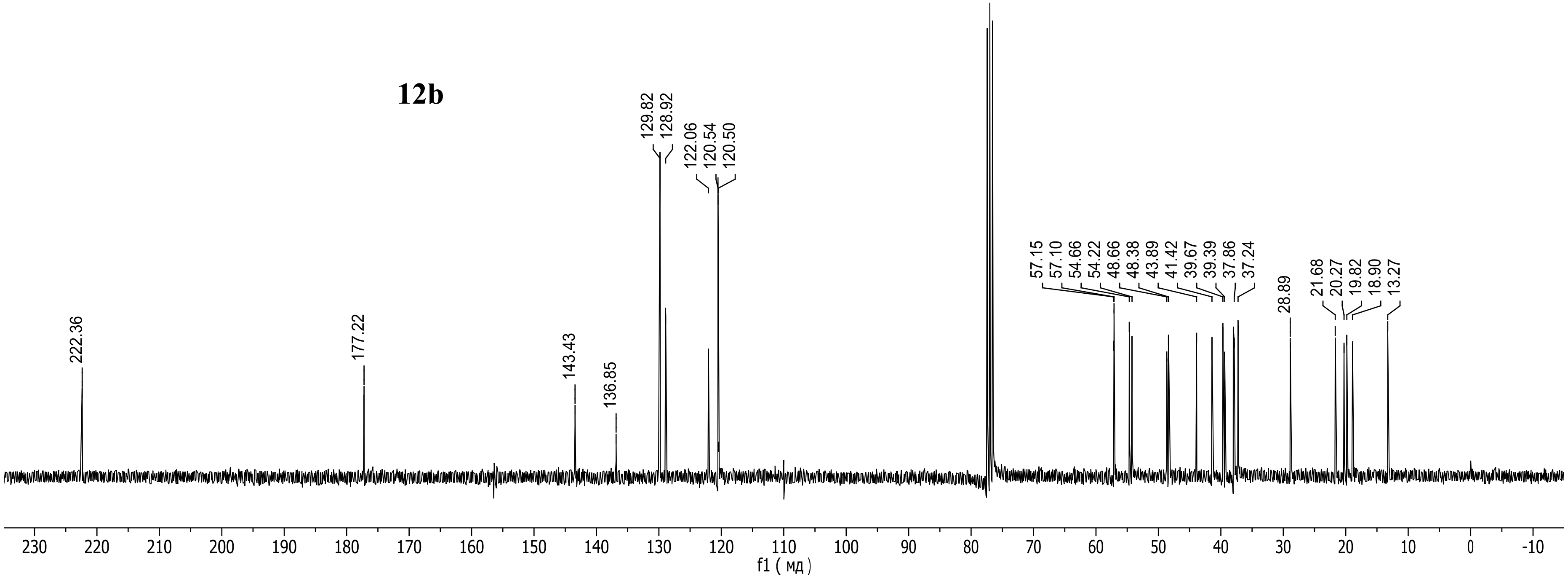

Figure S6. HRMS of compound **12b**.

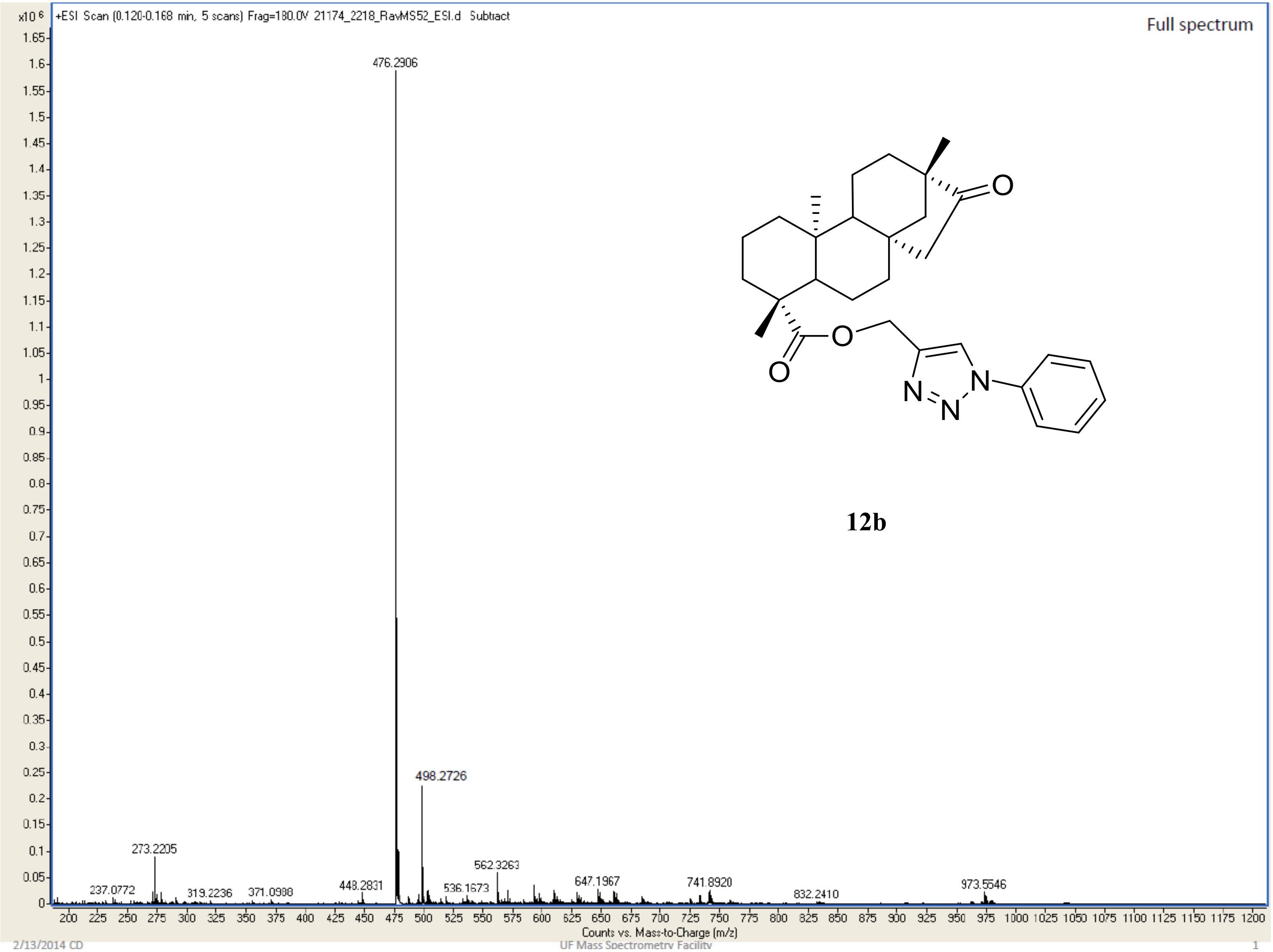

Figure S7. ^{1}H-NMR of compound **12c**.

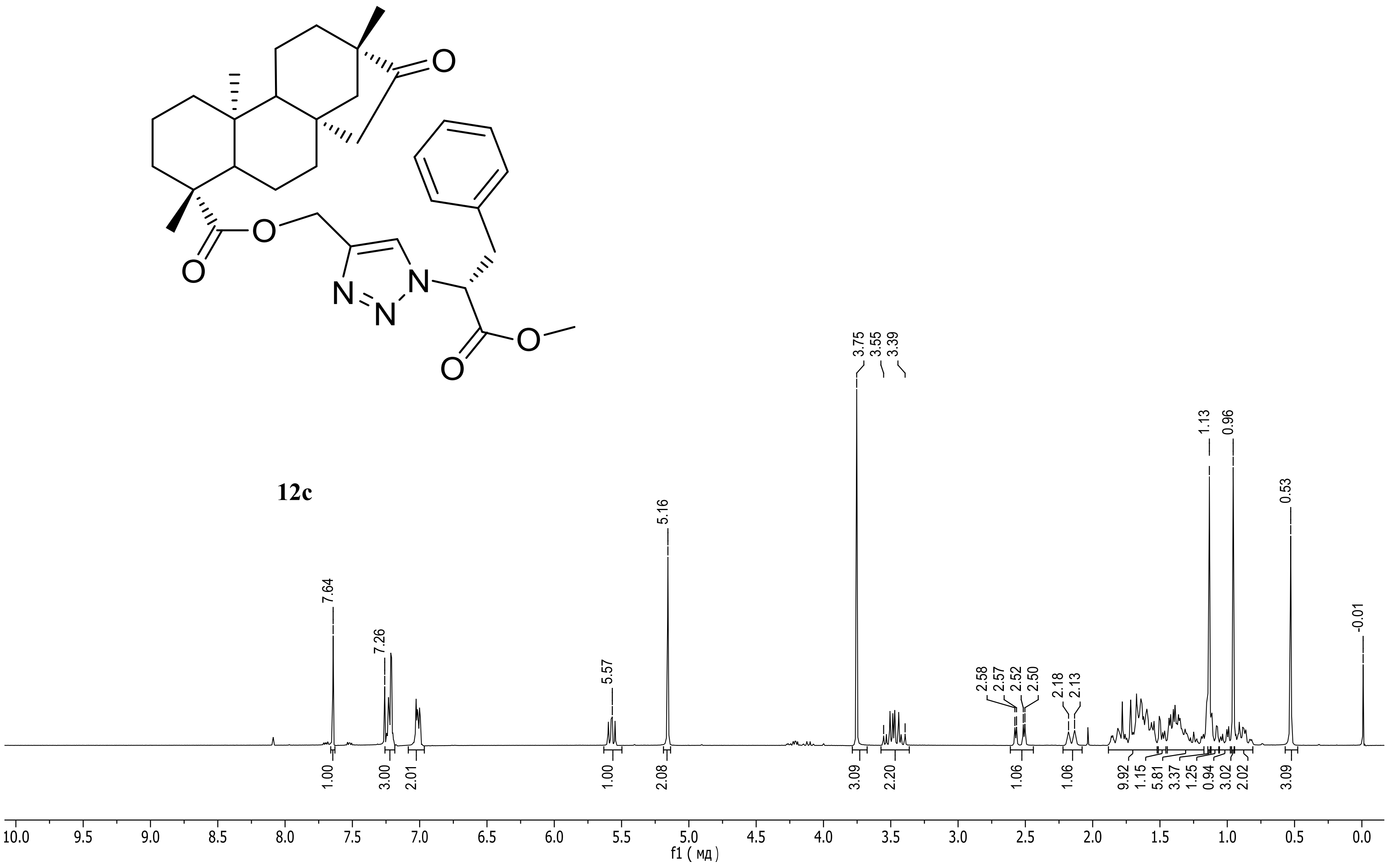

Figure S8. ^{13}C-NMR of compound **12c**.

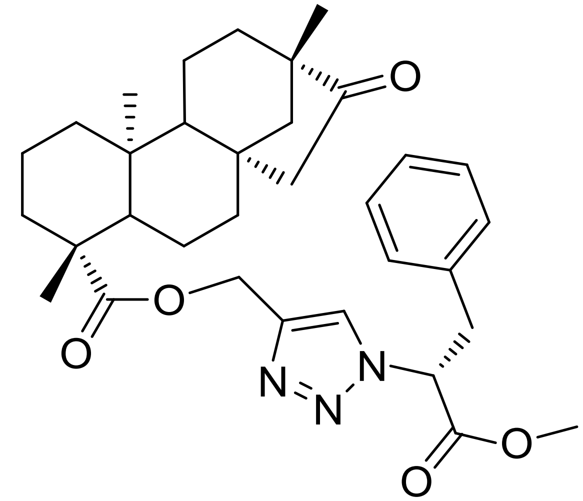

12c

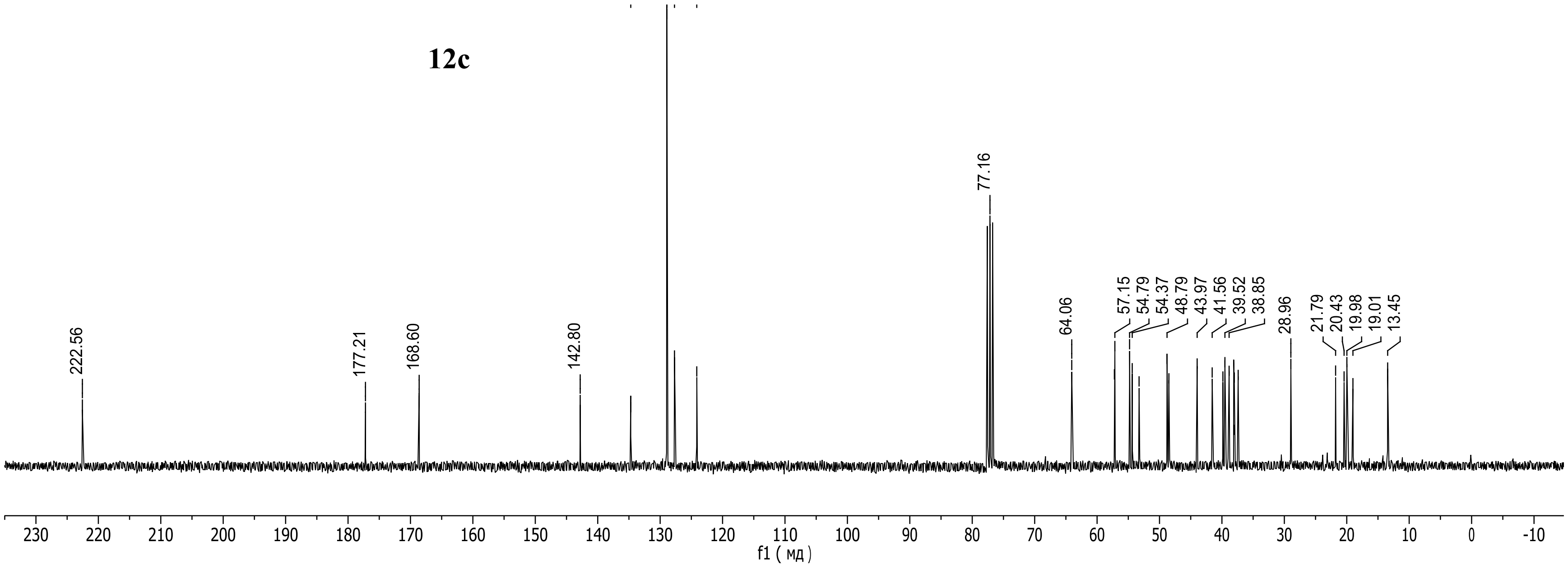

Figure S9. HRMS of compound **12c**.

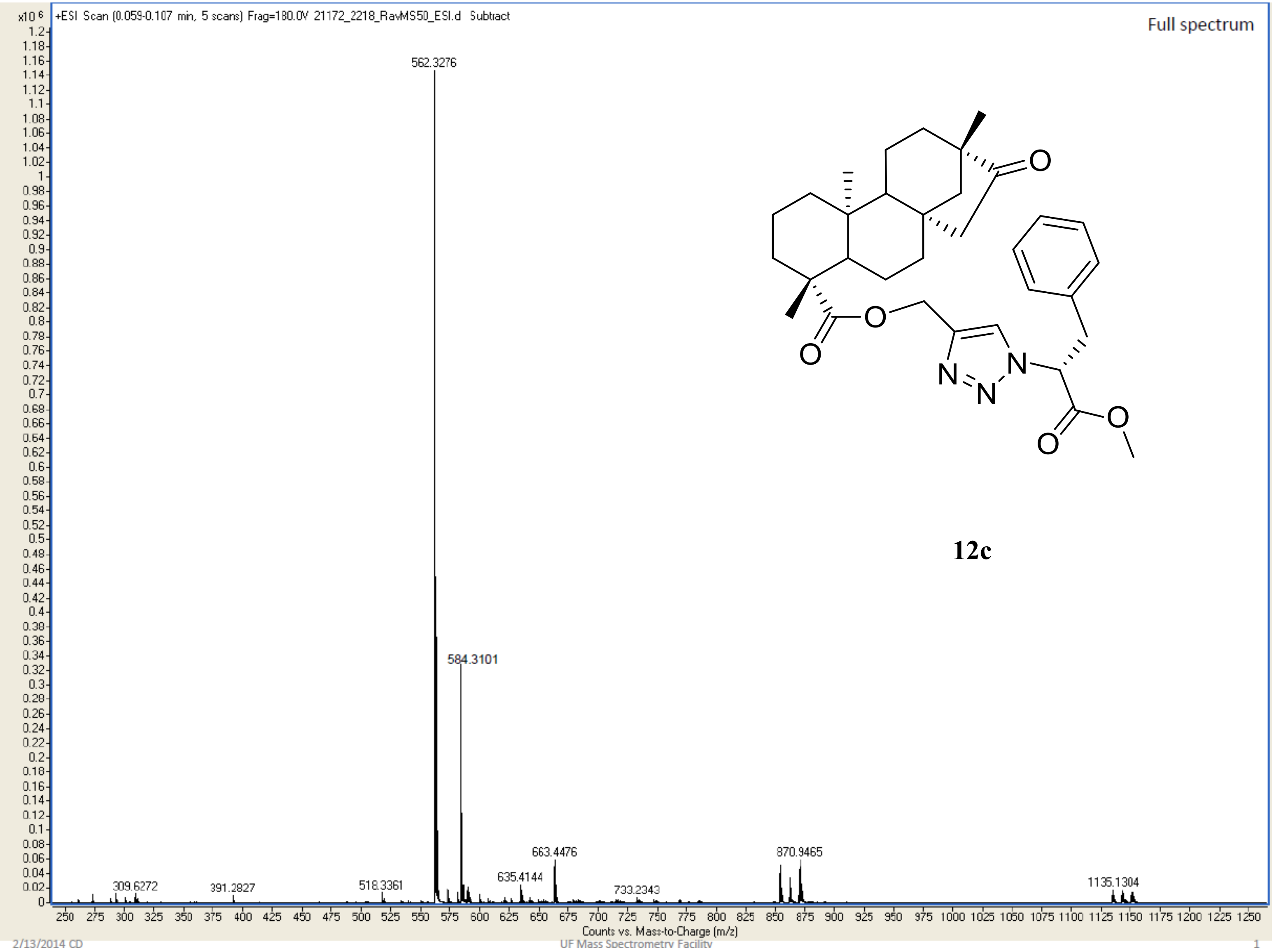

Figure S10. ^{1}H-NMR of compound **12d**.

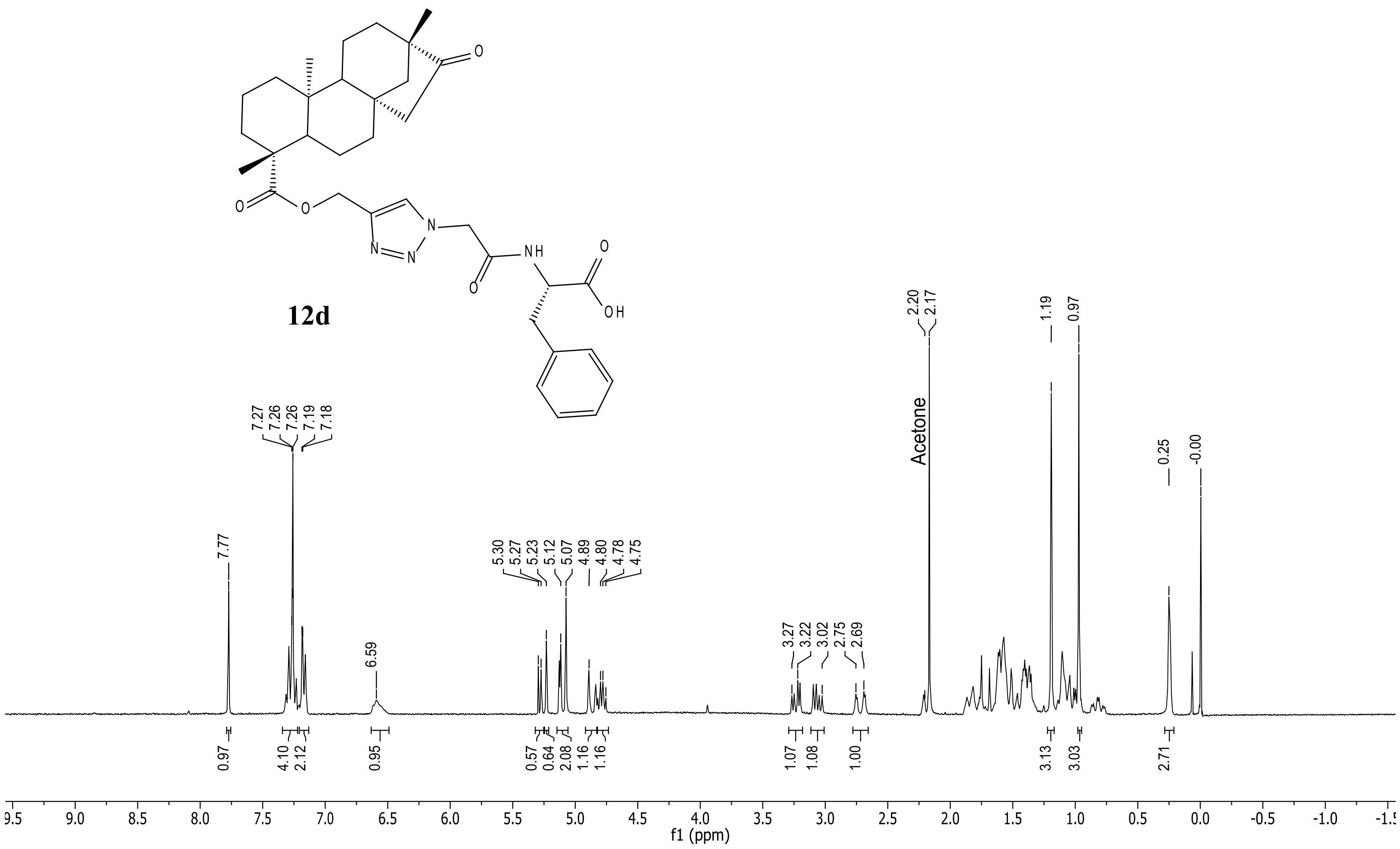

Figure S11. ^{13}C-NMR of compound **12d**.

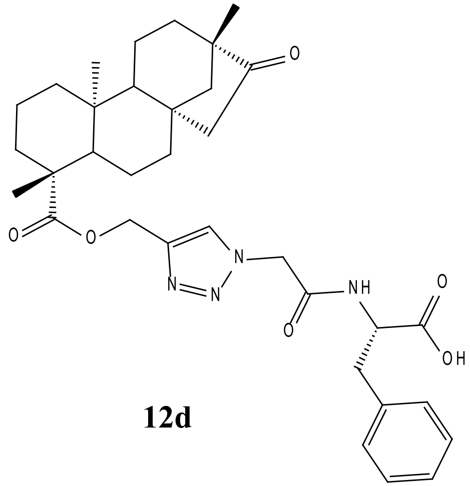

12d

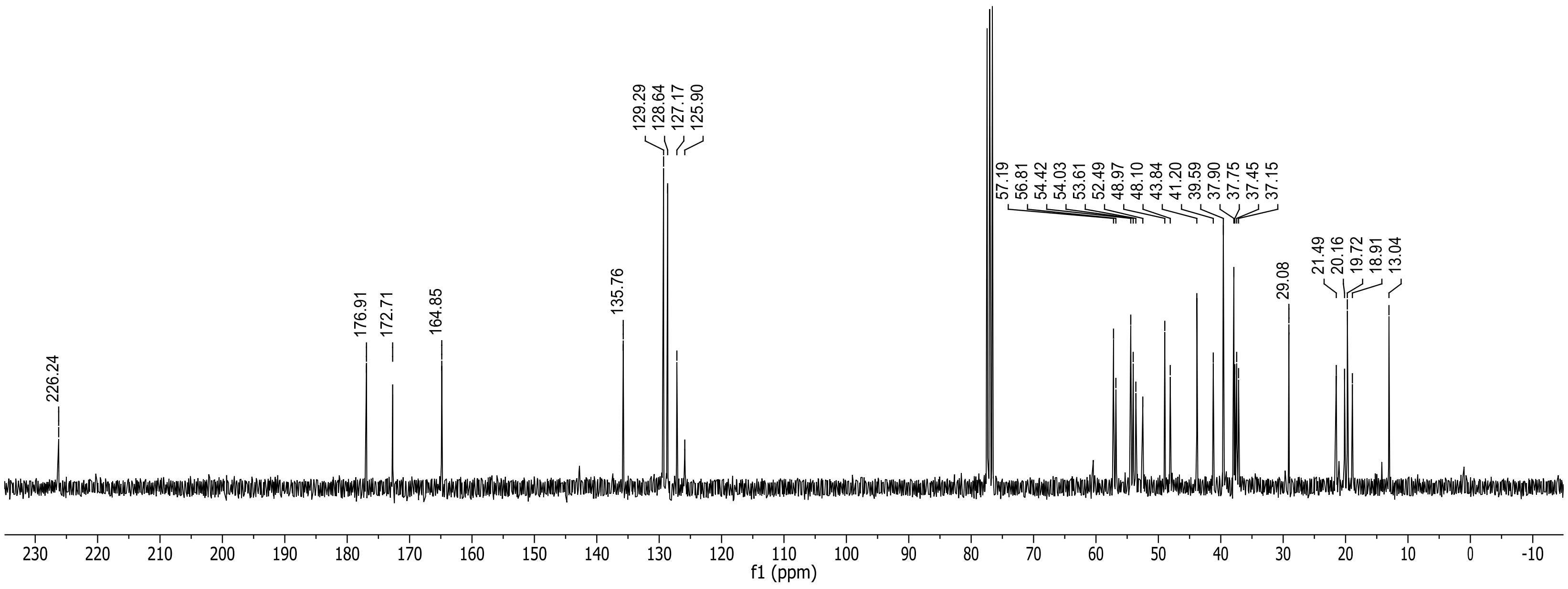

Figure S12. HRMS of compound **12d**.

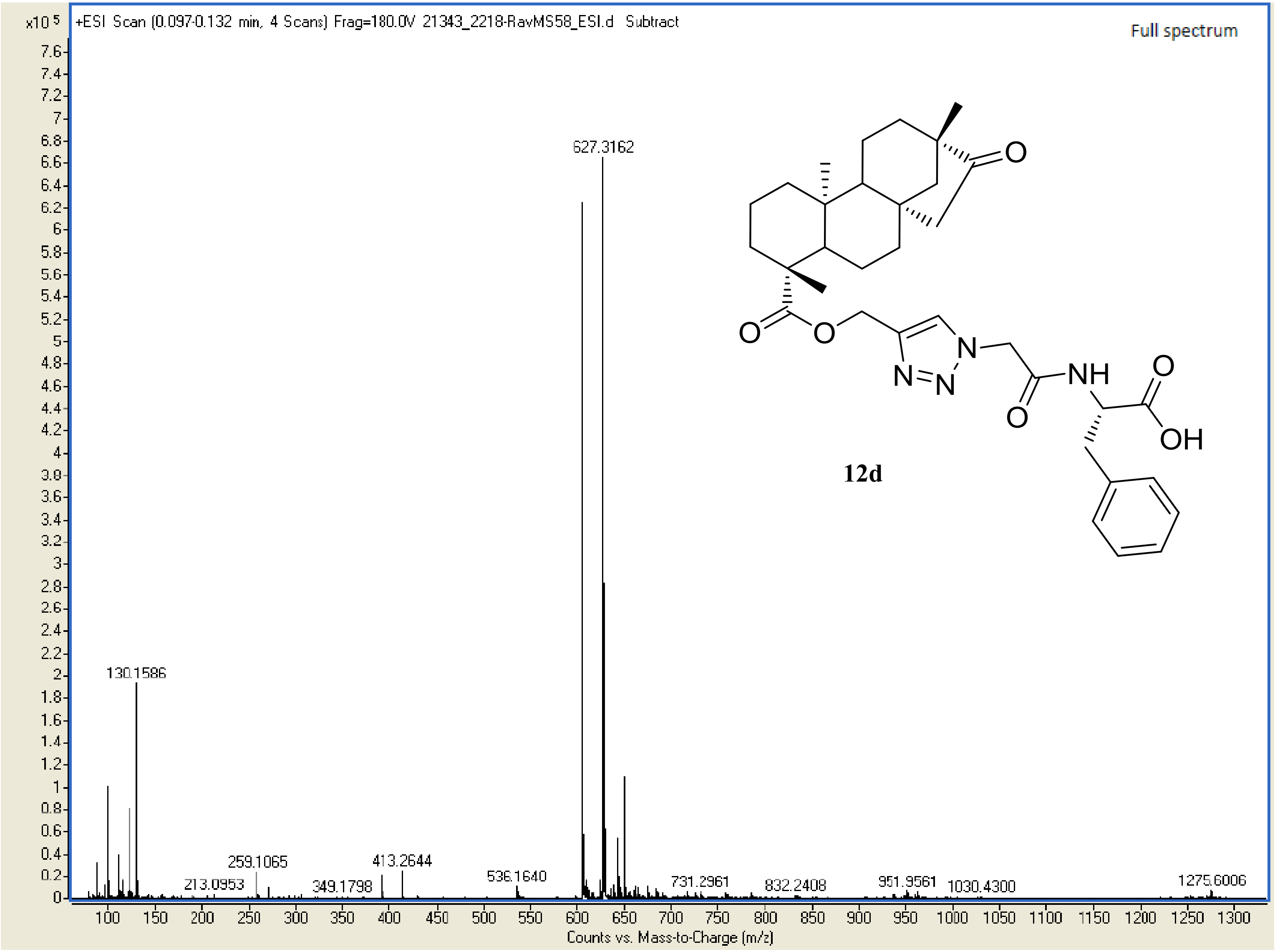

Figure S13. ^{1}H-NMR of compound **13a**.

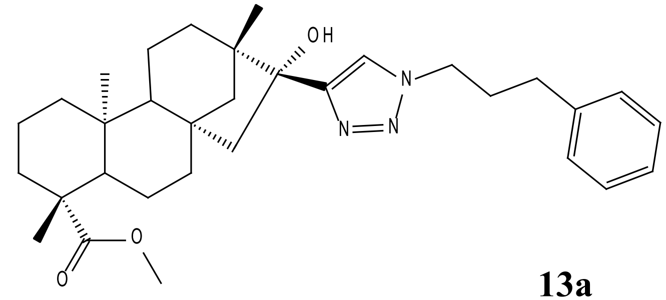

13a

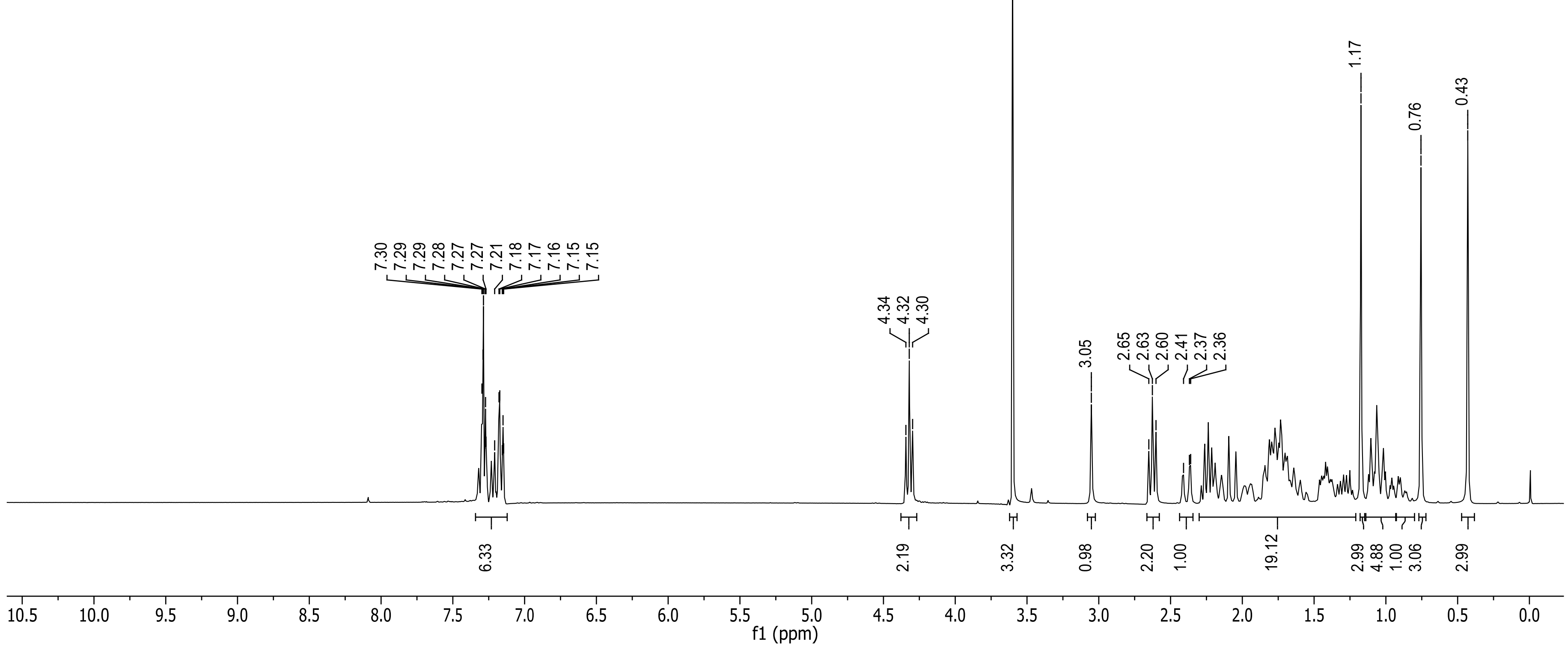

Figure S14. ^{13}C-NMR of compound **13a**.

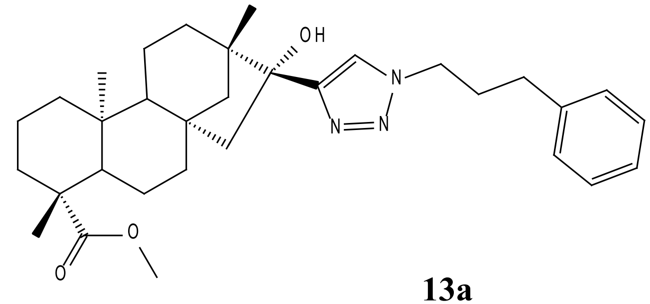

13a

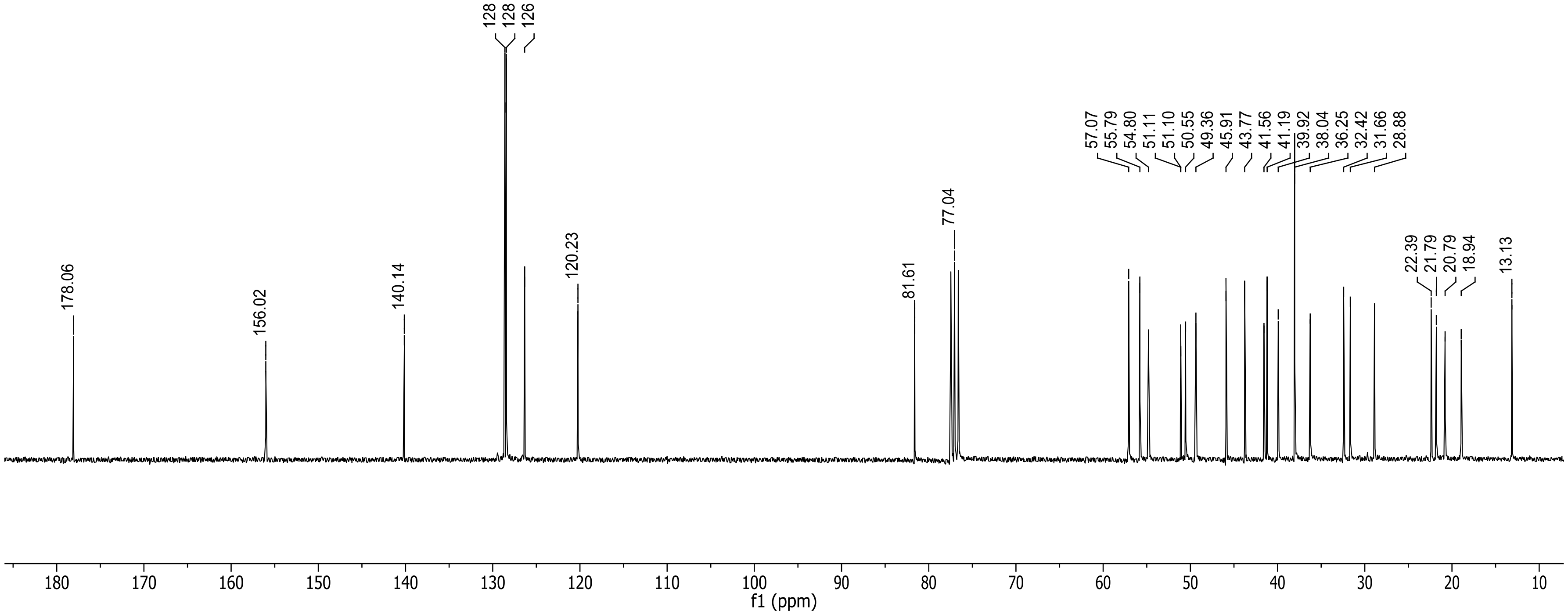

Figure S15. HRMS of compound **13a**.

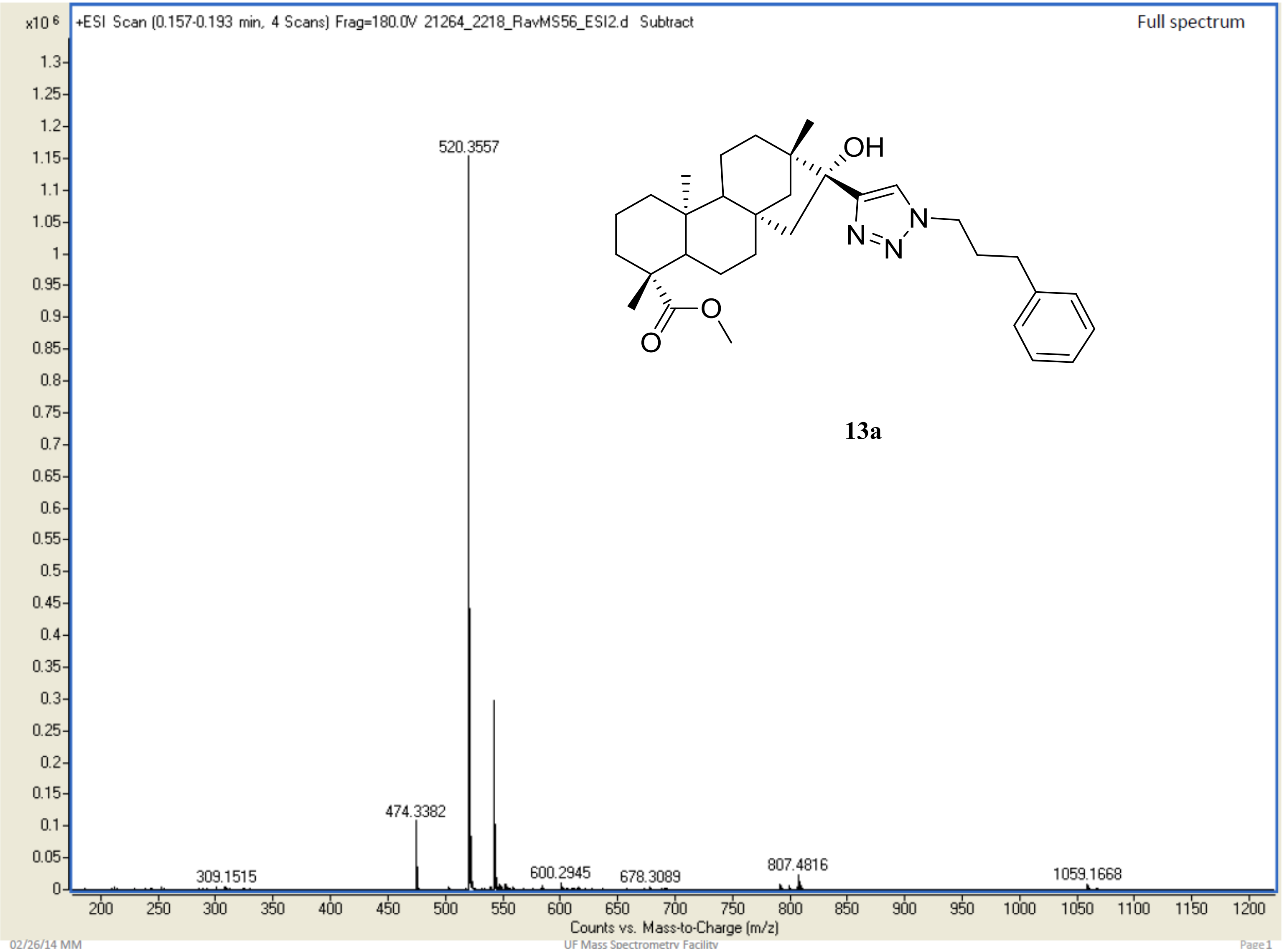

13a

Figure S16. ^{1}H-NMR of compound **13b**.

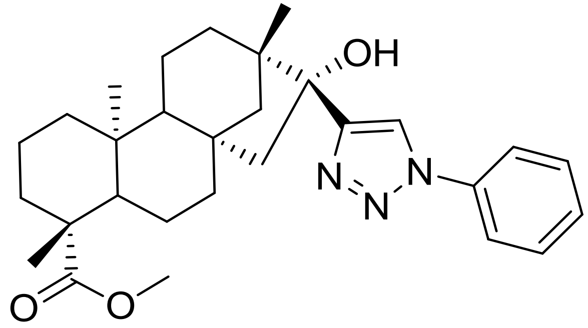

13b

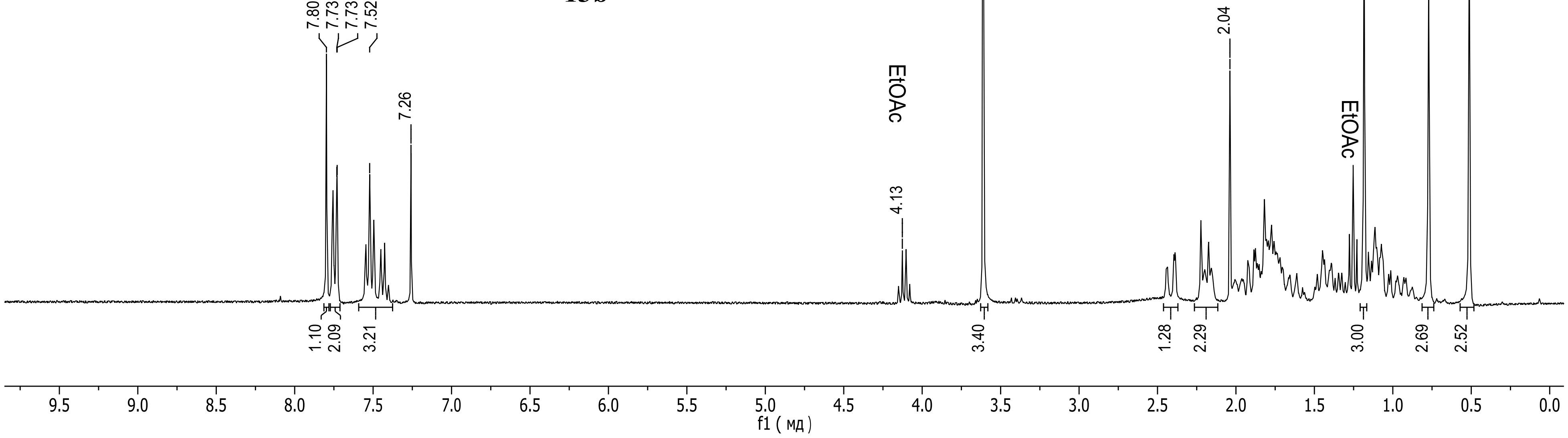

Figure S17. ^{13}C-NMR of compound **13b**.

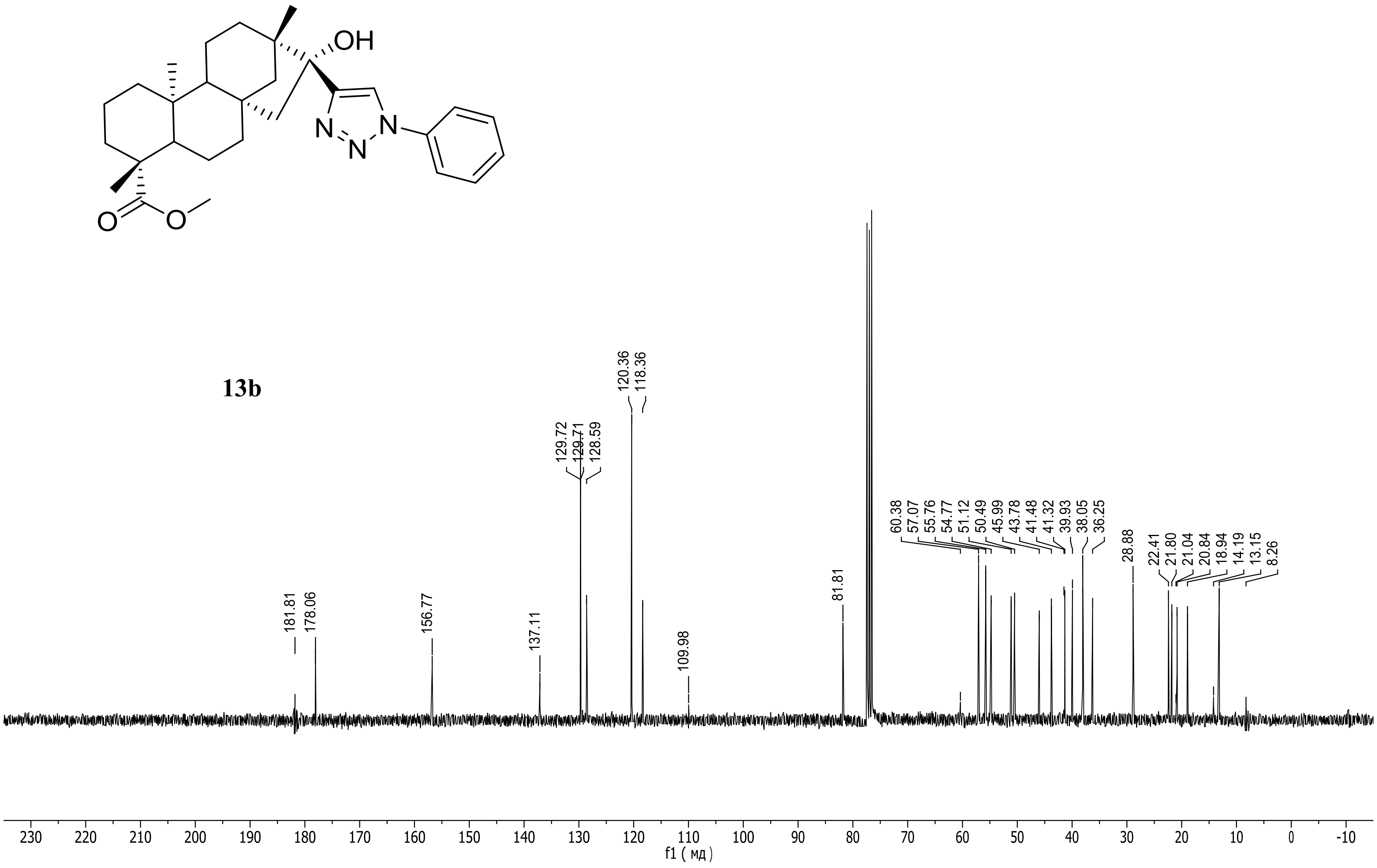

Figure S18. HRMS of compound **13b**.

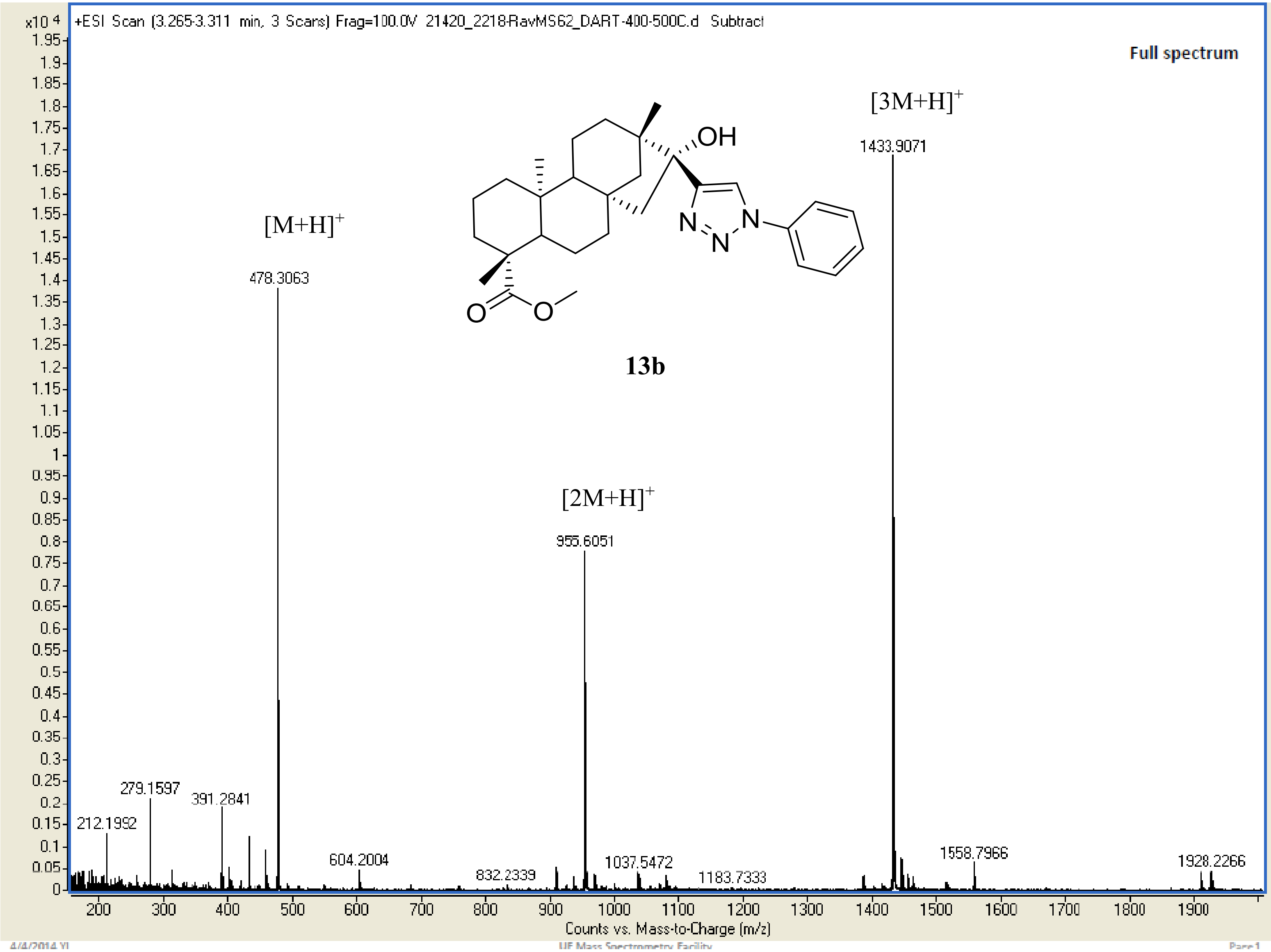

Figure S19. ^{1}H-NMR of compound **13c**.

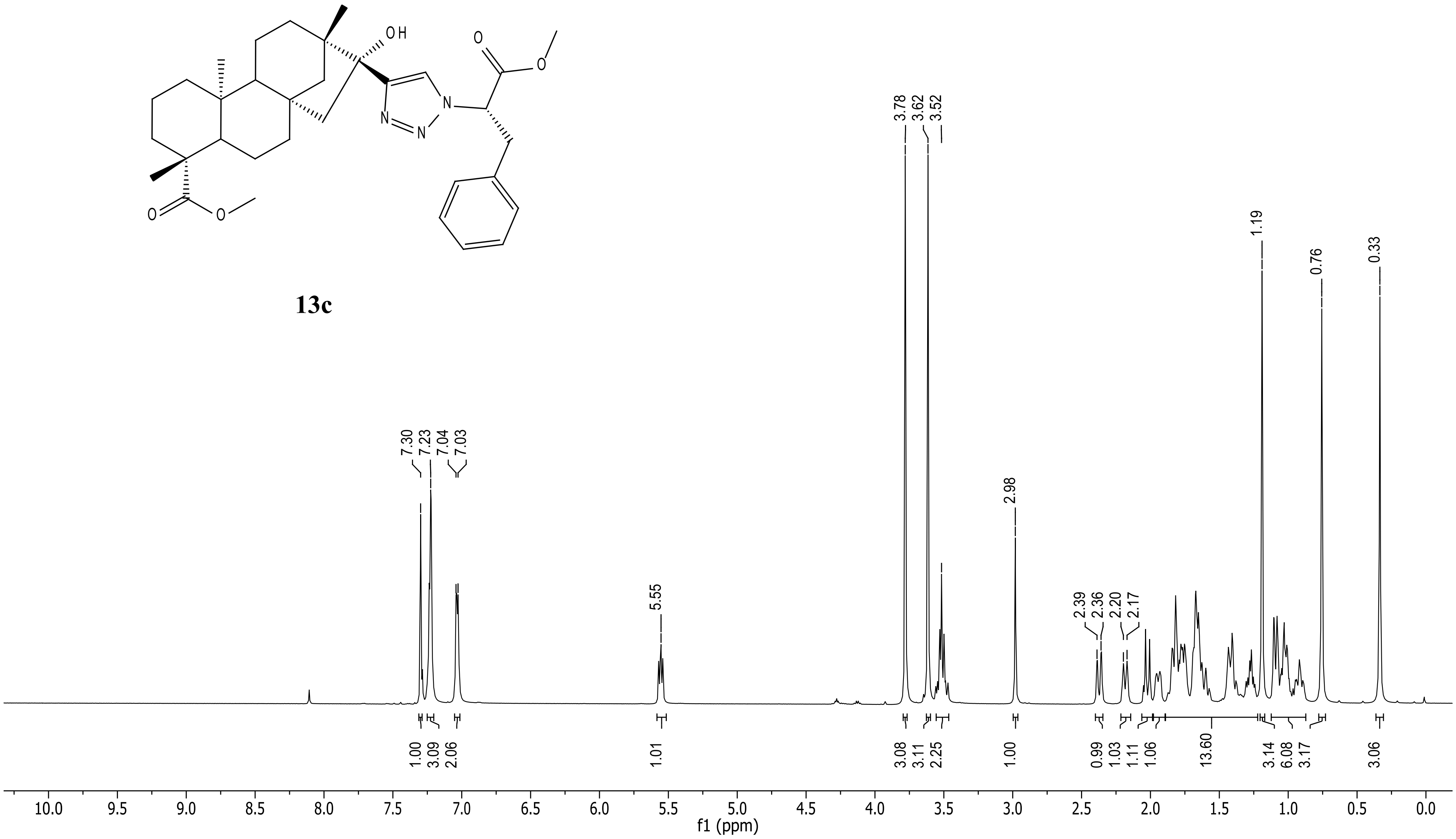

Figure S20. ^{13}C-NMR of compound **13c**.

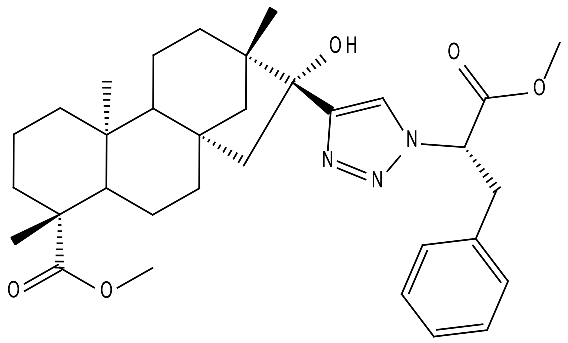

13c

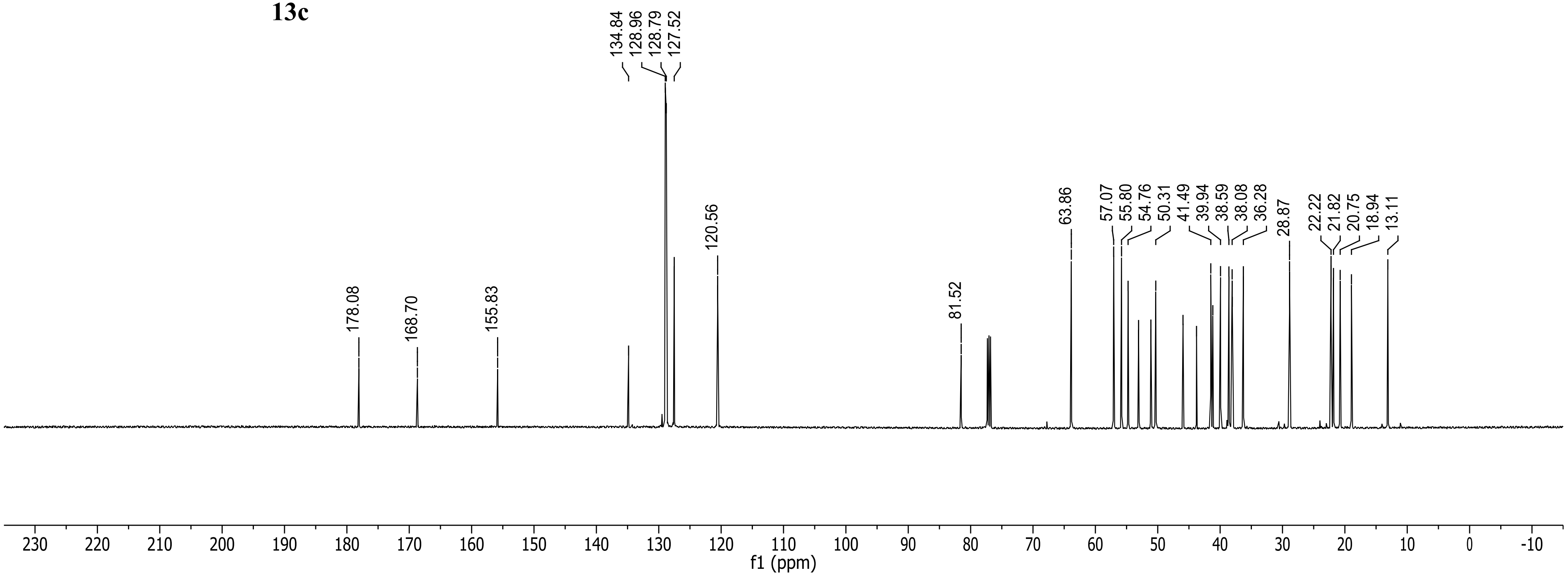

Figure S21. HRMS of compound **13c**.

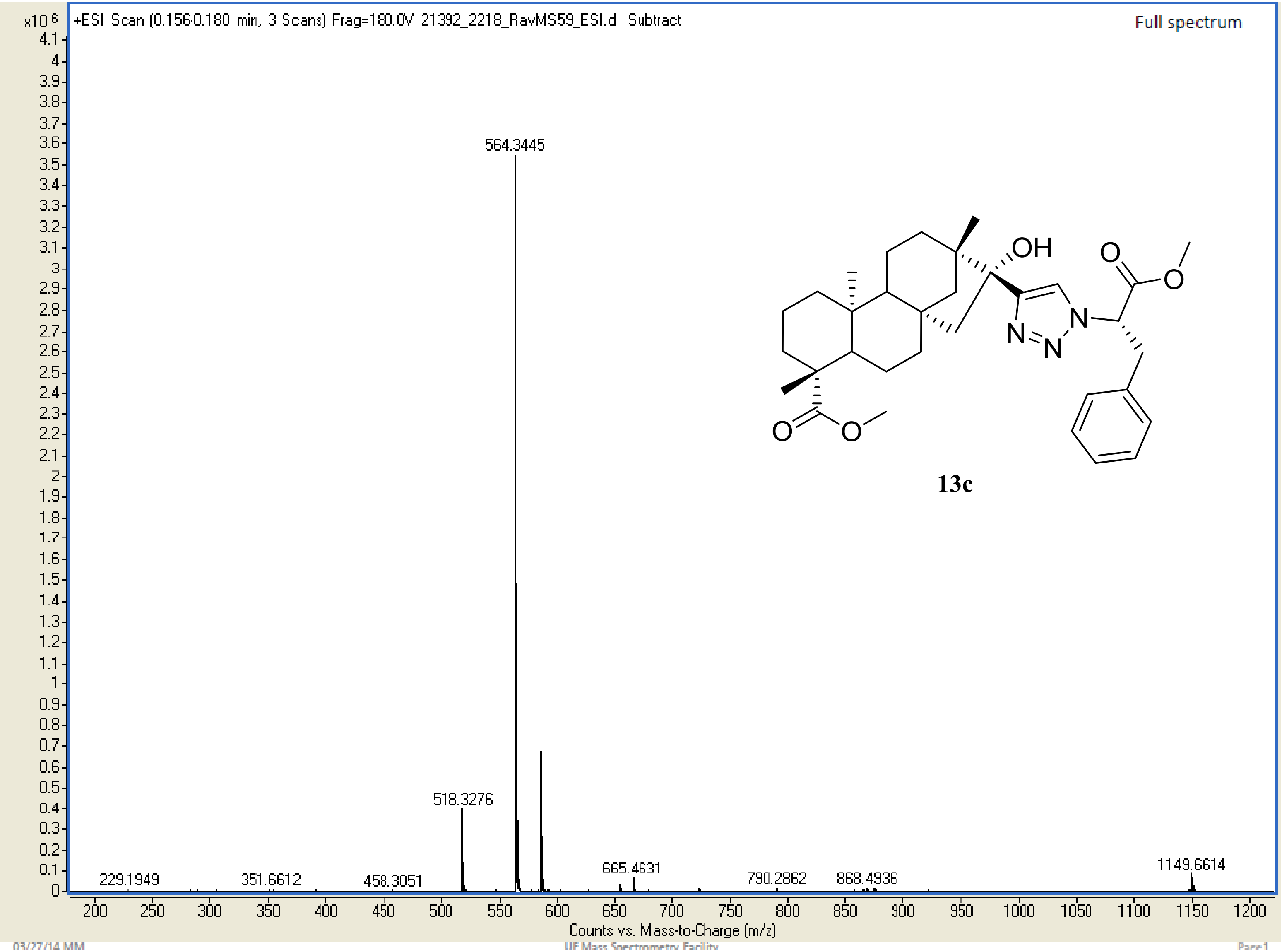

Figure S22. ^{1}H-NMR of compound **13d**.

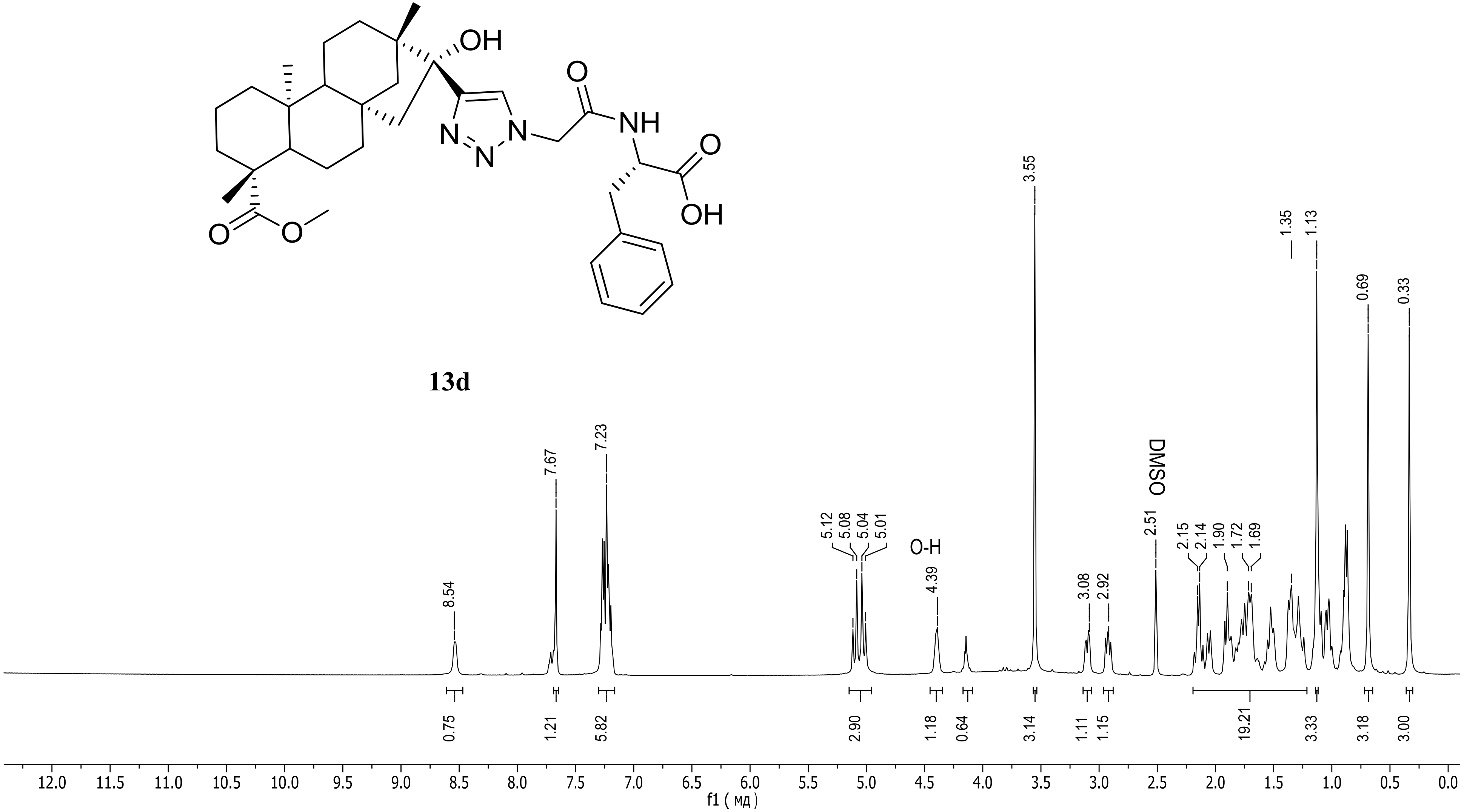

13d

Figure S23. ^{13}C-NMR of compound **13d**.

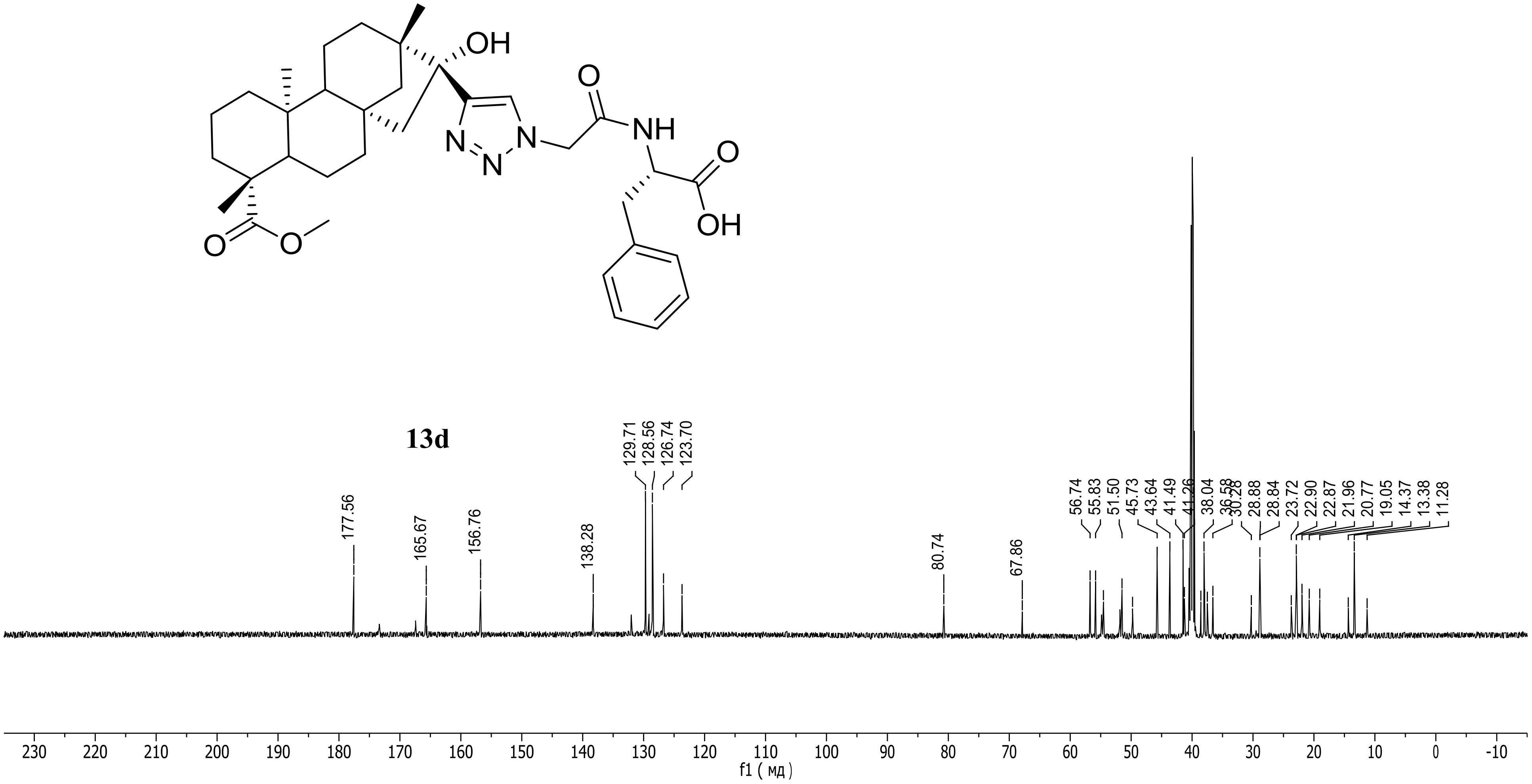

Figure S24. HRMS of compound **13d**.

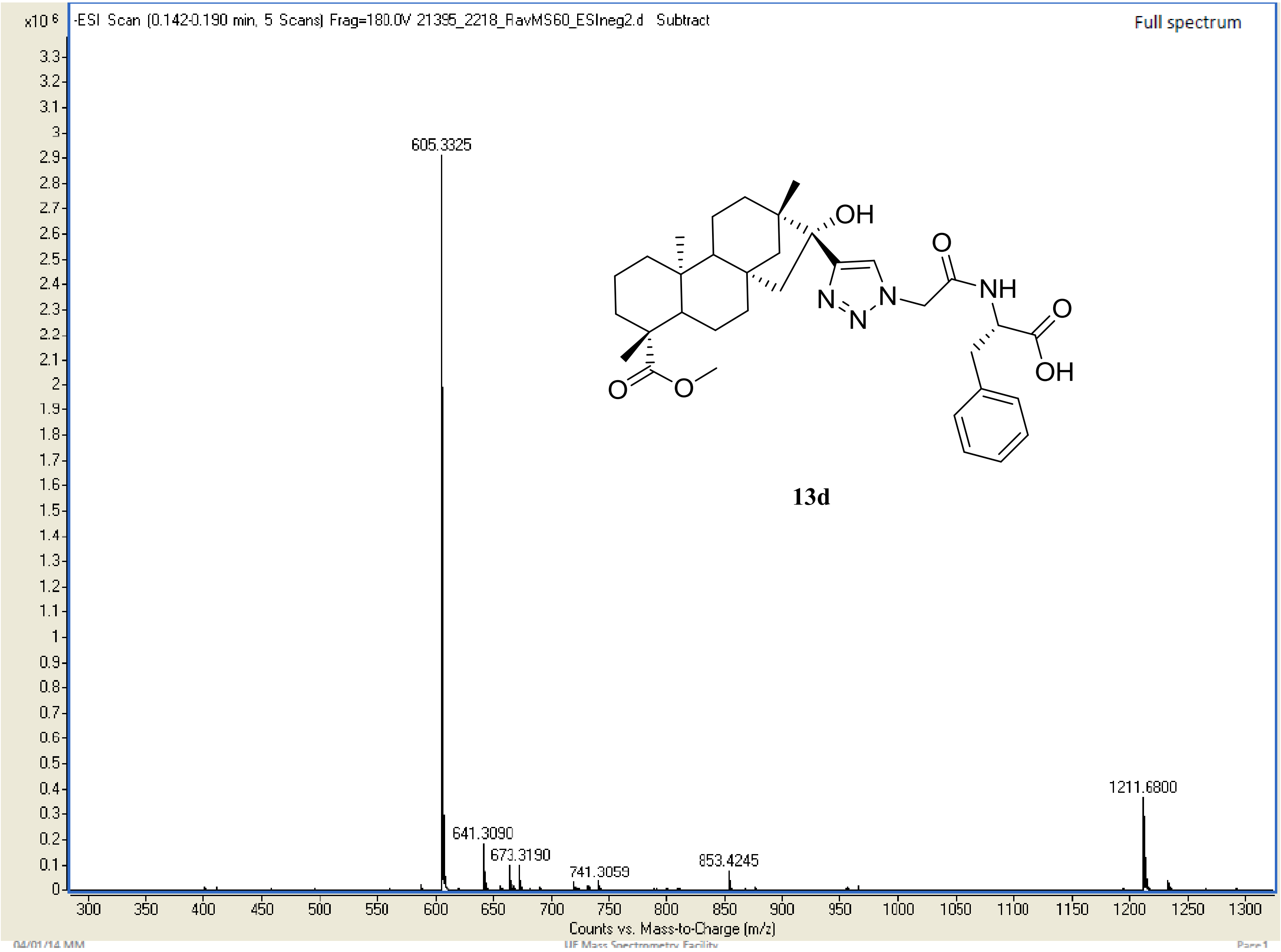

Supplement: Supplementary File 1 [file molecules-19-18676-s001.pdf]
